# Iron metabolic pathways in the processes of sponge plasticity

**DOI:** 10.1371/journal.pone.0228722

**Published:** 2020-02-21

**Authors:** Alexander D. Finoshin, Kim I. Adameyko, Kirill V. Mikhailov, Oksana I. Kravchuk, Anton A. Georgiev, Nicolay G. Gornostaev, Igor A. Kosevich, Victor S. Mikhailov, Guzel R. Gazizova, Elena I. Shagimardanova, Oleg A. Gusev, Yulia V. Lyupina

**Affiliations:** 1 N.K. Koltzov Institute of Developmental Biology, Russian Academy of Sciences, Moscow, Russia; 2 A.N. Belozersky Institute of Physical and Chemical Biology, Lomonosov Moscow State University, Moscow, Russia; 3 A.A. Kharkevich Institute for Information Transmission Problems, Russian Academy of Sciences, Moscow, Russia; 4 Lomonosov Moscow State University, Moscow, Russia; 5 Kazan Federal University, Kazan, Russia; 6 KFU-RIKEN Translational Genomics Unit, RIKEN National Science Institute, Yokohama, Japan; Cinvestav, MEXICO

## Abstract

The ability to regulate oxygen consumption evolved in ancestral animals and is intrinsically linked to iron metabolism. The iron pathways have been intensively studied in mammals, whereas data on distant invertebrates are limited. Sea sponges represent the oldest animal phylum and have unique structural plasticity and capacity to reaggregate after complete dissociation. We studied iron metabolic factors and their expression during reaggregation in the White Sea cold-water sponges *Halichondria panicea* and *Halisarca dujardini*. De novo transcriptomes were assembled using RNA-Seq data, and evolutionary trends were analyzed with bioinformatic tools. Differential expression during reaggregation was studied for *H*. *dujardini*. Enzymes of the heme biosynthesis pathway and transport globins, neuroglobin (NGB) and androglobin (ADGB), were identified in sponges. The globins mutate at higher evolutionary rates than the heme synthesis enzymes. Highly conserved iron-regulatory protein 1 (IRP1) presumably interacts with the iron-responsive elements (IREs) found in mRNAs of ferritin (FTH1) and a putative transferrin receptor NAALAD2. The reaggregation process is accompanied by increased expression of IRP1, the antiapoptotic factor BCL2, the inflammation factor NFκB (p65), FTH1 and NGB, as well as by an increase in mitochondrial density. Our data indicate a complex mechanism of iron regulation in sponge structural plasticity and help to better understand general mechanisms of morphogenetic processes in multicellular species.

## Introduction

The iron homeostasis is crucial for organisms relying on aerobic energy production, especially Metazoans [1,2]. The iron ions in a form of prosthetic groups are essential for key cellular processes such as genome replication and cell proliferation. Metabolism of iron and oxygen turnover are tightly connected and strictly regulated in animals. The iron-binding globin proteins are ubiquitous animal proteins that bind oxygen and participate in respiration and oxidative metabolism [3–6]. Multiple factors that regulate the iron and oxygen homeostasis in mammalian cells have been discovered in the last decades. The byproduct of respiration, singlet oxygen is a highly reactive radical that can damage different cellular structures and modulate intracellular signaling in different types of cells through specific protein photosensitizers [7–9]. The intracellular iron-containing photosensitizer protoporphyrin IX (PpIX) is the immediate precursor of heme, which serves as a cofactor to a functional core of proteins involved in storage and delivery of molecular oxygen. Excess of PpIX damages mitochondria [10,11], and its suppression is one of the major defense mechanisms against damages caused by singlet oxygen in mammalian tumors [12,13]. The outer mitochondrial membrane iron-sulfur protein MitoNEET controls intramitochondrial iron concentrations and allows tumor cells to tolerate reactive oxygen species and to avoid ferroptosis via activation of the PI3K/Akt/mTOR and COX2/AKT/ERK1/2 pathways [12,14,15]. At low oxygen availability, the hypoxia-inducible factors (HIFs) regulate the expression of genes that mediate cell’s adaptive responses connected to mitochondrial function, energy metabolism, oxygen binding and delivery [2,16–19]. The response to hypoxia and the ability to regulate oxygen requirements have been developed in the ancestral animals and intrinsically linked to the metabolism of iron and sulfur [20, 21]. The iron metabolism was intensively studied in mammalian species, whereas the data on iron pathways in evolutionary distant animals are limited.

Sponges (Porifera) are sessile filter-feeding animals and classically considered as the most basal lineage of Metazoa [22,23]. The sponge body structure is an aquiferous system used to pump and filter water for absorbing oxygen and nutrition, and in addition, it acts in the elimination of toxic compounds and for gamete release. The “ancient” sponges could participate in the ocean oxygenation via the redistribution of organic carbon by filtration [24]. The sponge cells have the capacity to transition between multiple cell types in a manner similar to the transdifferentiating stem cells [25]. This plasticity is utilized in the reaggregation of dissociated cells and the formation of multicellular aggregates after the sponge body dissociation [26–29]. Importantly, the sea sponges grow only in the presence of ferric ions [30]. In some species, the iron ions are involved in the formation of mineral elements of the skeleton—spicules. Bacterial symbionts of sponges can absorb iron, providing host cells with secondary metabolites [31,32]. The behavior of sea sponges in the sub-tidal zone involves water-filtering and avoiding damage by large particles upon sea tides and storm surges and should be adapted to seasonal changes in temperature and oxygen content.

In this report, we analyzed the iron metabolic pathways in two White Sea cold-water sponges from cl. Demospongia, *Halichondria panicea* (Pallas, 1766), which have spicules, and *Halisarca dujardini* (Johnston, 1842), which lack spicules. By using the transcriptome assembly and annotation, we obtained primary structures of proteins of the heme synthesis pathway, the iron-binding globins, the iron storage ferritin proteins, and key regulatory factors responsive to iron. The evolutionary trends in factors of iron pathways were analyzed by bioinformatics tools. Differential expression of genes analyzed by the RNA-Seq in *H*. *dujardini* sponge confirmed the involvement of the iron pathway factors in processes of the dissociation of the sponge body and the reaggregation of dissociated cells.

## Materials and methods

### Specimen collection

Specimens of the cold-water sea sponges *H*. *panicea* and *H*. *dujardini* were collected from the subtidal zone (0–2 m) at low-tide of the White Sea near the N.A. Pertsov White Sea Biological Station of Lomonosov Moscow State University (66°34′ N 33°08′ E). No specific permissions were required for the samplings, locations or activities. No sponge species were captured in a protected area, national park or private area, just as no protected or endangered species were involved in the study.

The water temperature at the time of collection was 0+5°C (March and November) and +15°C (July). The sampling was done in a way that the sponges remained attached to the substrate (alga), allowing sponge regeneration. Sponges were kept in aquariums (5 l, natural seawater, 6–8°C or 10–12°C) and transported to the Koltzov Institute of Developmental Biology (Moscow, Russia). Sponges were placed individually in 5 l aquariums with seawater (6–8°C or 10–12°C) under a 12 h light/12 h dark cycle using artificial light sources. Sponges were acclimated under these conditions for one week prior to experiments. We used 10 specimens of *H*. *panicea* collected in March 2017, 10 specimens of *H*. *dujardini* collected in July 2017, and 10 specimens of *H*. *dujardini* collected in November 2017.

The use of sponges in the laboratory does not raise any ethical issues, and therefore approval from regional and local research ethics committees is not required. The field sampling did not involve endangered or protected species. In accordance with local guidelines, the permissions for collection of material were not required.

### Sponge body dissociation and reaggregation procedures

In order to exclude possible effects of the mineralization processes (spicule formation), the dissociation/reaggregation experiments were carried out only with sponge *H*. *dujardini*. The specimens for these experiments were collected in November when the water oxygenation is at a maximum. Sponges were dissociated and cultivated individually in the filtered seawater (FSW) sterilized with the Millex-GP syringe filter units 0.22 μm (Merck Millipore, USA). Sponge body was cut in pellets by small-sized scissors or scalpel in a small Petri dish with FSW. The tissue was pelleted five to eight times and resuspended in 1 ml FSW by using a microdispenser with a 1000-μl tip. The sample was then resuspended in 4 ml FSW with a 5-ml glass pipet and divided into 1-ml portions. After the addition of 4 ml FSW to each portion, the samples were resuspended by the pipet and filtered through a 40-μm nylon mesh and then centrifuged for 5 min at 300 *g* and 10–12°C. The dissociated cells were analyzed within 30 min after dissociation. The total number of live cells and percentage (usually more than 96–98%) were calculated by using a standard hemacytometer in 10-μl portions mixed with 10 μl of 0.4% trypan blue. In reaggregation experiments, the dissociated cells in FSW were diluted to the concentration of 1×10^6^ cells/ml and cultivated in 6-well plates (2 ml per dish) at 10–12°C. The cell aggregates were analyzed at 24 h after tissue dissociation. Each cell culture was monitored for cell viability by microscope Leica DM RXA2 (Leica, Germany) equipped with a digital camera OLYMPUS and Leica DMR Software.

### RNA isolation

Total RNA was isolated from the sponge body, cell suspensions or cell aggregates with a TRI Reagent (Molecular Research Center, Inc.) according to the manufacturer’s instructions.

### cDNA library construction, quality detection, and Illumina sequencing

Extracted RNA was treated by Dnase I (Ambion, Thermo Fisher Scientific, USA) according to the DNA-free^™^ Kit (Ambion, Thermo Fisher Scientific, USA) protocol. For pair-end sequencing 1 μg of RNA was depleted using Ribo-zero rRNA Removal Kit (Human / Mouse / Rat) (Illumina, Inc). The effectiveness of this procedure was monitored using the Pico mRNA protocol on the Agilent Bioanalyzer 2100 (Agilent Technologies, USA). The cDNA library of each sample was constructed using NEBNext^®^ Ultra^™^ II Directional RNA Library Prep Kit for Illumina^®^ (New England Biolabs, UK) according to the manufacturer’s instruction. The cDNA library quality and fragment length distributions were verified using an Agilent 2100 DNA High Sensitivity Kit. The sequencing of the resulting cDNA libraries was carried out on an Illumina Hiseq2500 instrument with paired-end 125-bp reads. For 50 bp single-end Illumina Hiseq2500 sequencing, mRNA was isolated using NEBNext^®^ Poly(A) mRNA Magnetic Isolation Module (New England Biolabs, UK) from 300 ng of total RNA and cDNA library was constructed as described above. The average number of the read pairs per sample was 48M with the total number of 291M for 6 samples of *H*. *dujardini* and 146M for 3 samples *H*. *panicea* (Table 1). The average number of single-end reads per sample was 30M with the total number of 241M reads for 8 samples of *H*. *dujardini* (S1 Table).

**Table 1:** Total number of paired-end reads per sample used for *de novo* transcriptome assemblies.

#	Organism	Sample	Collection Time	# of reads
1	*H*.*dujardini*	Cells	Nov.	47,776,964
2		Tissue	Nov.	47,635,615
3		Aggregates	Nov.	50,429,456
4		Tissue	Jul.	50,015,085
5		Cells	Jul.	48,176,886
6		Frozen tissue	Jul.	47,838,658
7	*H*.*panicea*	Frozen tissue	Mar.	48,449,710
8		Tissue	Mar.	48,513,985
9		Cells	Mar.	49,882,301

### Transcriptome assembly and annotation

All paired-end reads were pooled for each organism and processed as follows. First, reads were trimmed by the quality and cleaned from adapter sequences using trimmomatic v. 0.36 [33] with parameters “ILLUMINACLIP:TruSeq3-PE.fa:2:30:10 SLIDINGWINDOW:4:5 LEADING:5 TRAILING:5 MINLEN:25”, recommended by the authors of the Trinity software. Correctly paired reads were further subjected to kmer-based error-correction using Rcorrector v1.0.3.1 [34] with “-k 31” parameter. The processed reads for each species, *H*. *dujardini* and *H*. *panicea*, were used for *de novo* transcriptome assembly (Table 1) using Trinity v.2.8.4 [35] with default parameters, except for *in silico* reads normalization, which was turned off. Obtained assemblies were then assessed with transrate v. 1.0.3 [36]. Transcripts were queried with diamond-blastx v.0.9.24.125 [37] against the uniclust90 database [38] and with *hmmer v*.3.2.1 [39] against the PFAM v. 32 database [40]. Trinity’s Transdecoder v. 5.5.0 was used along with these search results to predict probable coding sequences, which were clustered afterward using CD-HIT v.4.6.8 [41] with a 90% identity threshold to remove possible assembly artifacts. The completeness and possible contamination of the resulting proteome were then checked with BUSCO v.3 [42]. Along with basic annotation provided for Transdecoder, we used Trinotate v.3.1.1 [43], KEGG Automatic Annotation Server [44] and custom blastp searches against proteins of the Demospongiae species for extended annotation.

### Differential expression analysis for *H*. *dujardini* dissociated and reaggregated cells

Single-end reads for the three states: intact tissue, dissociated cells, and aggregates with 3, 3 and 2 replicates, respectively, were mapped to the transcriptome assembly and the read counts were quantified with RSEM v.1.3.1 [45]. The average percentage of unaligned reads per sample was 3.6% (S1 Table). The expression correlation matrix was plotted to check the expression consistency between samples (S1 Fig). Replicates tissue#3 and cells#3 were excluded from the downstream analyses since they deviated significantly from their counterparts. All downstream analyses were made on the ‘gene’, not ‘isoform’ level. We did not use external utilities for re-clustering ‘isoforms’ to ‘genes’, since recent studies did not show that they significantly outperform Trinity’s own algorithm [46]. The R package *edgeR* [47] was used to analyze differential expression between the states. We followed major pipeline suggested by the authors for an analysis of more than 2 groups, namely: (1) filtering out low-expressed genes with *filterByExpr()* function (using default parameters and no manual CPM threshold) with following library size recalculation; (2) TMM-normalization; (3) BCV estimation; (4) fitting GLM Quasi-Likelihood model; (5) testing genes for any difference in ‘cells vs. tissue’ and ‘aggregates vs. tissue’ contrasts; (6) BH-correction of p-values and using 0.001 as a threshold for resulting FDR; and (7) computing corrected CPM values with *cpm()* function. The R package *ComplexHeatmap* [48] was used to visualize differential expression for curated gene sets. Differential expression analysis was then carried out using the standard *edgeR* pipeline. 119,409 out of 357,155 Trinity’s ‘genes’ were left after filtering out low expressed ones. Remaining genes were tested for any difference in ‘cells vs. tissue’ and ‘aggregates vs. tissue’ contrasts. Using standard FDR threshold of 0.05, we obtained that 43% of genes were differentially expressed (39% if at least 2-fold change is required). Therefore, we used a stricter FDR threshold of 0.001 for visualization.

### Functional annotation and phylogenetic analysis of iron metabolism proteins

Amino acid sequences for a curated set of 10 proteins of heme biosynthesis pathway were retrieved for 28 reference organisms with the custom script from the KEGG orthology database [49]. Orthologous sequences from the *H*. *dujardini* and *H*. *panicea* proteomes were added to the set, and the sequences were aligned using clustalo v.1.2.4 [50] with default parameters. Pairwise distance matrices were obtained from these alignments using PROTDIST tool v.3.69 from PHYLIP package [51] with default parameters (Jones-Taylor-Thornton matrix, no gamma distribution of rates among positions). Guide tree topology was inferred from the Open Tree of Life project [52] via *rotl* package for R [53]. These alignments along with the guide tree were provided to Erable software v.1.0 [54] in order to determine relative divergence rates and branch lengths.

Protein domains were found with CDVist web-server [55], the respective trees were constructed with IQ-TREE [56]. Visualization of trees and protein domains was made with ete-toolkit [57]. Visualization of protein alignments with secondary structure elements was made with ESPript web-server [58]. Large sequence alignments were visualized with Jalview2 [59]. Iron-responsive elements (IREs) in sponge mRNAs were predicted and visualized by SIREs web-server [60].

### Western-blotting assay

Cell lysates of the *H*. *dujardini* tissue, cells and cell aggregates were extracted in RIPA buffer and protease inhibitor (Sigma). Aliquots containing 80 μg of protein were diluted in sample buffer and maintained for 4 min in a water bath at 95°C. SDS-PAGE electrophoresis (160V) in 12% polyacrylamide gel was then performed, followed by protein transfer to a 0.45-μm nitrocellulose membrane. The membranes were incubated with 5% non-fat milk for 1 h and then incubated overnight at 4°C with the following primary antibodies and titrations: rabbit anti-pALAD (polyclonal, middle-region (ARP41657_P050), Aviva Systems Biology, Canada) 1:500; rabbit anti-FTH1 (polyclonal,#4393, Cell Signaling, USA) 1:500; mouse anti-β-actin (monoclonal, ACTBD 11B7, Santa Cruz Biotechnology, Santa Cruz, USA) 1:5000. Incubation for 1.5 h with secondary anti-mouse and anti-rabbit IgG HRP conjugated antibodies (GE Healthcare Life Solutions, Boston, USA) was performed, and the blot was immersed in ECL luminol enhancer solution (GE Healthcare Life Solutions, Boston, USA). Optical densities of bands on the X-ray film were analyzed using the standard program ImageJ. At first, we drew graphs of the dependence of optical density on the total protein amount subjected to Western blotting. For further experiments, we used the protein amounts for which the dependence was linear on the graph. Results were normalized against β-actin content in samples.

### ALAD and FTH1 immunofluorescence assay

The immunofluorescent staining of ALAD with the polyclonal anti-pALAD antibody (ARP41657_P050, Aviva Systems Biology, Canada) and the polyclonal FTH1antibody (4393, Cell Signaling, USA) in the *H*. *dujardini* samples was used for the assessment of the subcellular localization of these proteins. The cells were mounted on the glasses and fixed by 4% paraformaldehyde solution (on filtered seawater) for 2–4 h and then were consecutively incubated in 0.5% Triton X-100 with 5% fetal bovine serum in PBS for 40 min at 20°C; then with the first polyclonal antibodies to ALAD or FTH1 (1: 500) prepared in PBS with addition of 0.5% Triton X-100 and 5% FBS for 18 h at 8–10°C; then they were washed for 5 min in PBS and incubated with the second antibodies: Alexa Fluor 488 goat anti-rabbit IgG (Invitrogen, USA) (1: 700) for FTH1 or Alexa Fluor 594 donkey anti-rabbit IgG (Invitrogen, USA) (1: 800) for ALAD, prepared in PBS with addition of 0.3% Triton X-100 and 5% fetal bovine serum for 2 h at 20°C. The Hoechst-33342 (Invitrogen, USA) staining for nucleus was done for 10 min after incubation with the second antibodies. Then the slides were washed in PBS and placed in Mowiol (Calbiochem, Germany) to be analyzed using a Leica DM RXA2 fluorescent microscope (Germany). We obtained fluorescence images of the cells by using fluorescence microscopy (Leica DM RXA2) at λexc = 495 nm and λem = 517 nm for FTH1 as well as at λexc = 551 nm and λem = 573 nm for ALAD. The specificity of the first antibodies was confirmed by using control experiments in which the procedure was performed in their absence.

### Analysis of mitochondrial density

The method for estimation of mitochondrial density was adapted from a study of Rosin et al. (2018). Mitochondria were stained in the *H*. *dujardini* samples by using the MitoTracker Green kit (Invitrogen, Carlsbad, CA, USA) (500 nM, 60 min incubation) and visualized by means of fluorescence microscopy with filters at λexc = 495 nm and λem = 517 nm (Leica DM RXA2 fluorescent microscope, Germany). The fluorescence area of each cell was contoured by using a drawing tool of the Image J software. Next, the integrated density (contoured area × mean gray value) calculated by the software was annotated. The mean fluorescence background was measured by selecting five different regions of the field without cells and calculating the mean gray value. The corrected total cell fluorescence (relative fluorescence unit) was obtained by the following formula: CTFC = Integrated density − (contoured area × mean fluorescence background).

### Statistical analyses

The non-parametric data on mitochondrial density was presented as median, minimum, and maximum values. A comparison between the group samples was performed using Mann-Whitney’s test. All other data were parametric and shown as mean and standard deviation. Comparisons between the groups were performed by using the ANOVA variance test, followed by Tukey’s test. The level of significance was set to 1%.

## Results

### Sequencing and assembling de novo transcriptomes

**Fig 1:**
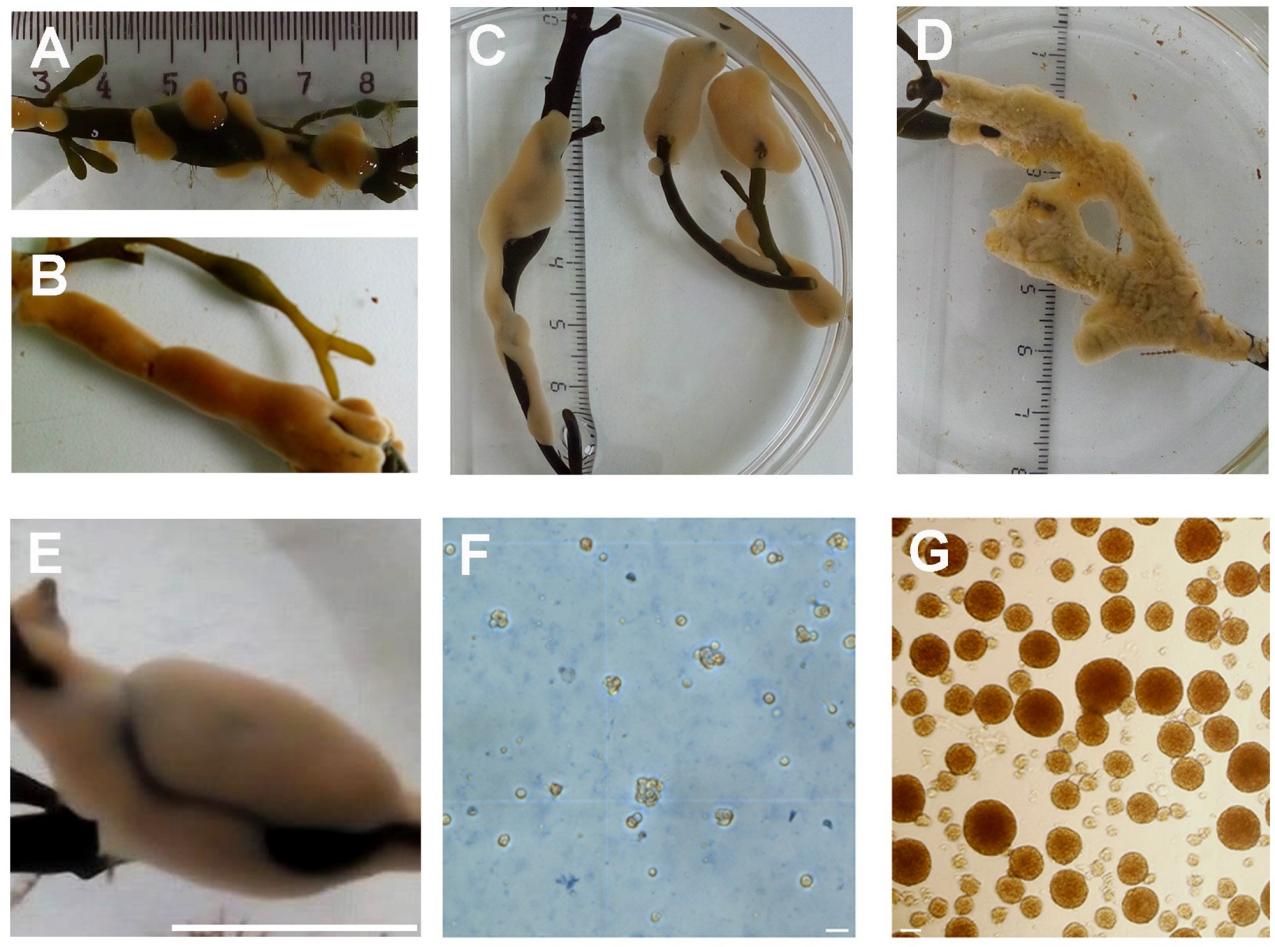
The specimens of the sponges *H*. *dujardini* and *H*. *panicea*. *H*. *dujardini*: **(A, B)** July tissues; **(C, E)** November tissues, scale–100 mm; **(F)** dissociated cells, scale–10 μm, (**G)** aggregated cells, scale–10 μm, *H*. *Panicea*: **(D)** March sample.

The samples of cold-water sea sponges *H*. *dujardini* and *H*. *panicea* collected in different seasons are shown in Fig 1(A)–1(E), and the dissociated and aggregated cells of *H*. *dujardini* in Fig 1(F) and 1(G).

In order to characterize the metabolic pathways involved in the sponge body regeneration, we performed a transcriptomic analysis of sponge cells in different morphological states.

The data were deposited to the National Center for Biotechnology Information (NCBI) for Halisarca dujardini (PRJNA594150) and Halichondria panicea (PRJNA594151).

The numbers of paired-end reads per sample obtained are shown in Table 1. The average number of read pairs per sample was 48M, totaling 291M for the 6 samples of *H*. *dujardini* and 146M for the 3 samples of *H*. *panicea*. The paired reads pooled from the libraries of each species that passed the adapter trimming and post-filtering (99.7%) were used to construct *de novo* transcriptome assemblies.

The statistics of the resulting transcriptomes are summarized in S2 Table. More than 90% of core Metazoan BUSCO genes were detected in both assemblies (91.92% and 97.55% for *H*. *dujardini* and *H*. *panicea*, respectively), with less than 3% found as fragments (2.76% and 1.53%), revealing that sequencing was sufficiently deep (S3 Table). Although bacterial and fungal BUSCOs were also prominently present in the assemblies, no additional filtering was carried out, since all the transcripts of interest were manually verified with blast searches against the Demospongiae or Metazoan sequences databases. Transcripts of *H*. *dujardini* were additionally verified with our draft genome of this species. Expression estimates for *H*. *dujardini* were obtained in a separate single-end RNA-Seq experiment, and the majority of those reads (94–98%) were successfully aligned to the transcriptome assembly (S1 Table). The assembled sequences were deposited in the GenBank for both studied sponge species *H*. *dujardini* and *H*. *panicea*.

### Iron processing in sponges

**Fig 2:**
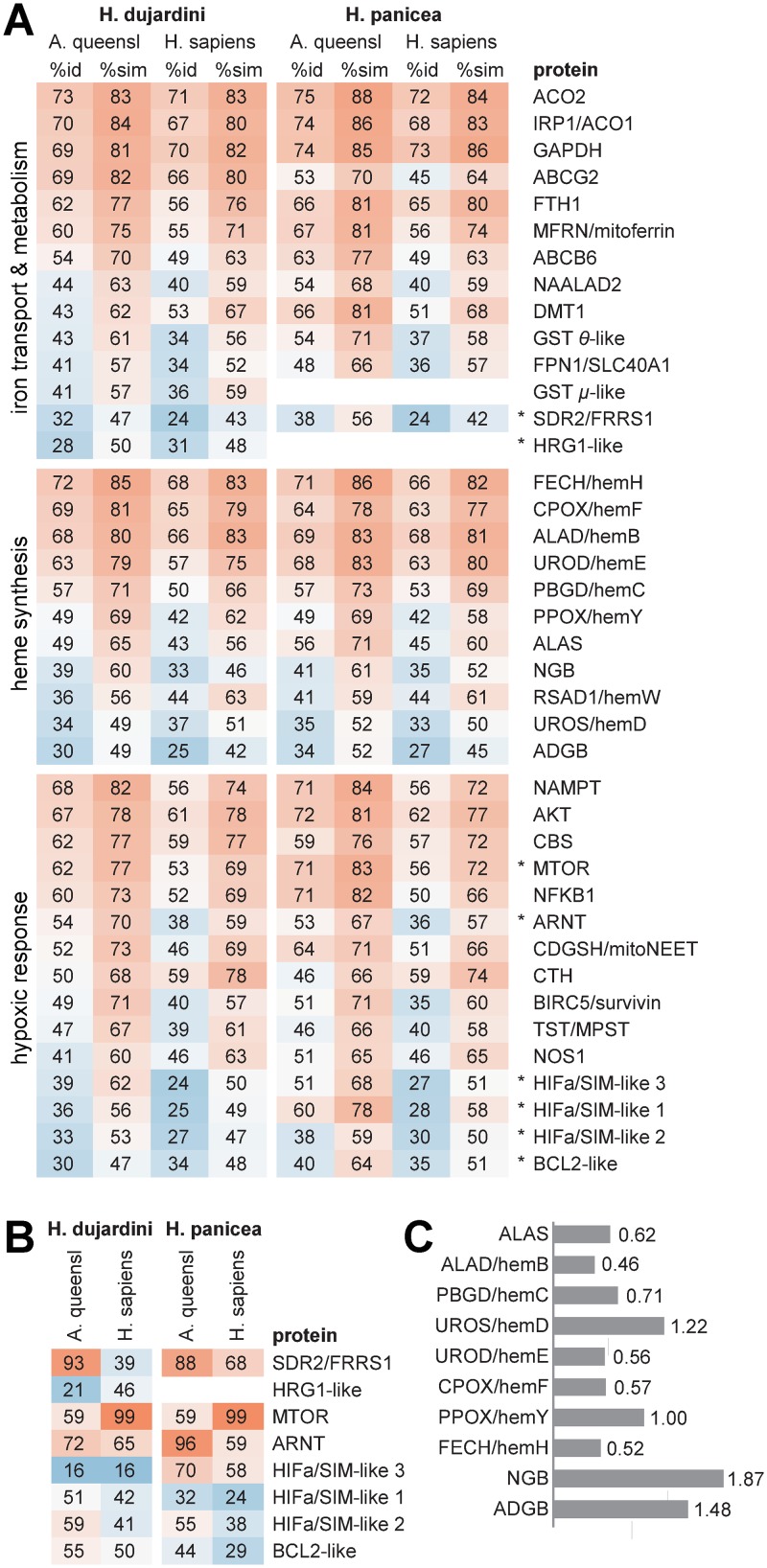
Homology features for iron metabolic proteins of sponges. **(A)** Identity and similarity percentage values for proteins of *H*. *dujardini* and *H*. *panicea* w.r.t. their human and *A*. *queenslandica* orthologs obtained with blastp (for the accession numbers see S4 Table). A. queensl, *A*. *queenslandica*; stars, sequences with query-per-subject coverage less than 70%. **(B)** Percentage of query-per-subject alignment coverage for the sequences marked in (A) obtained with blastp. **(C)** Relative rates of sequence divergence for ten proteins involved in heme synthesis and processing, estimated by Erable software using distance matrices for 30 eukaryotic species including sponges *H*. *dujardini*, *H*. *panicea* and *A*. *queenslandica*. Distance matrix was computed for each protein using its amino acid sequence alignment.

Using the *de novo* transcriptome assemblies, we recovered sponge homologs of proteins involved in the heme biosynthesis and transport, iron metabolism and response to hypoxia. The homology features and the accession numbers of corresponding proteins of *H*. *dujardini*, *H*. *panicea* and their human and *Amphimedon queenslandica* orthologs are presented respectively in Fig 2(A) and 2(B) and S4 Table. The majority of discovered amino acid sequences featured complete ORFs and lengths comparable to the ones of their orthologs, which implied high query-per-subject alignment coverage (exceptions are indicated in Fig 2B).

**Fig 3:**
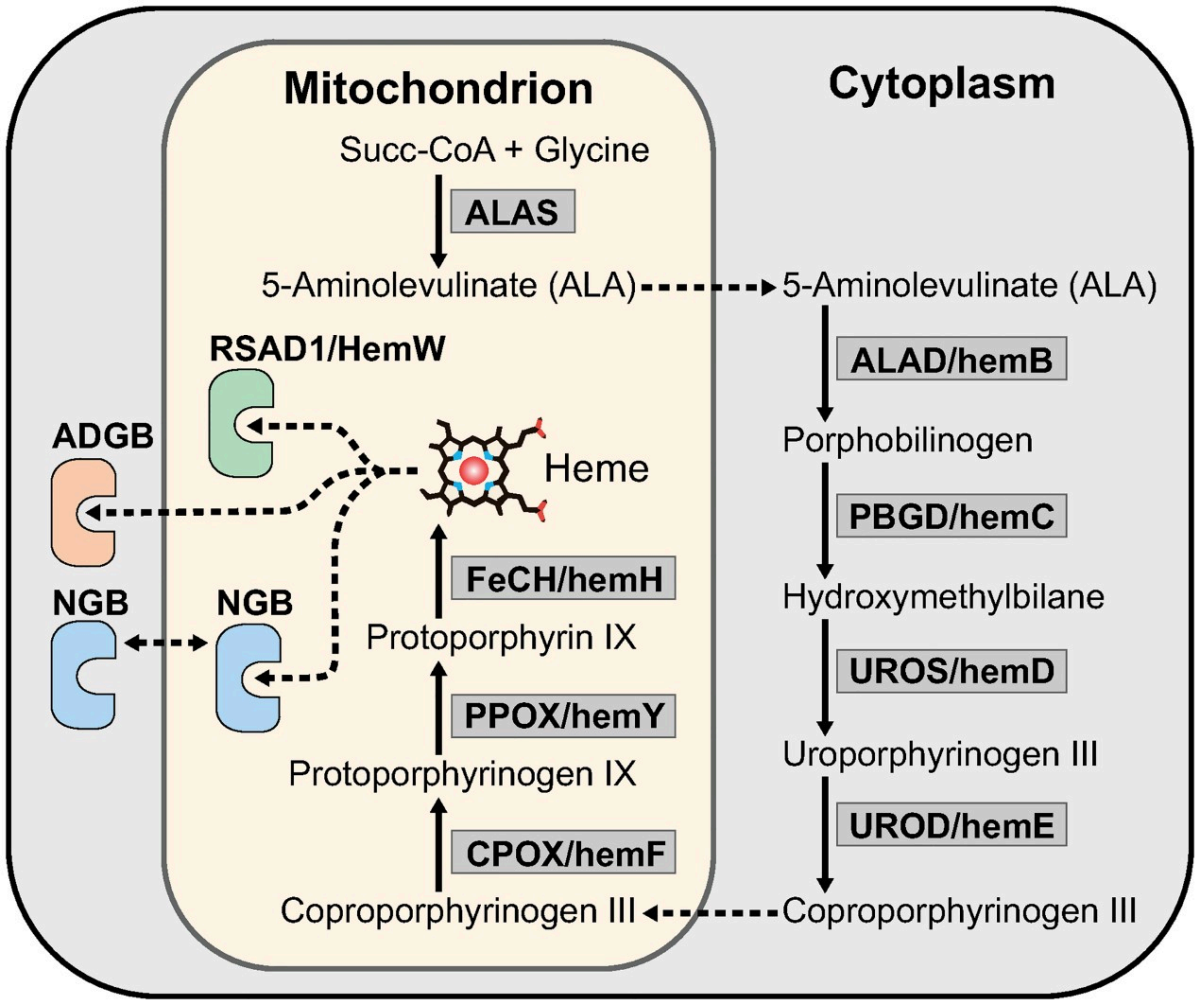
The predicted heme biosynthesis pathway in sponges (modified from [61]).

**Fig 4:**
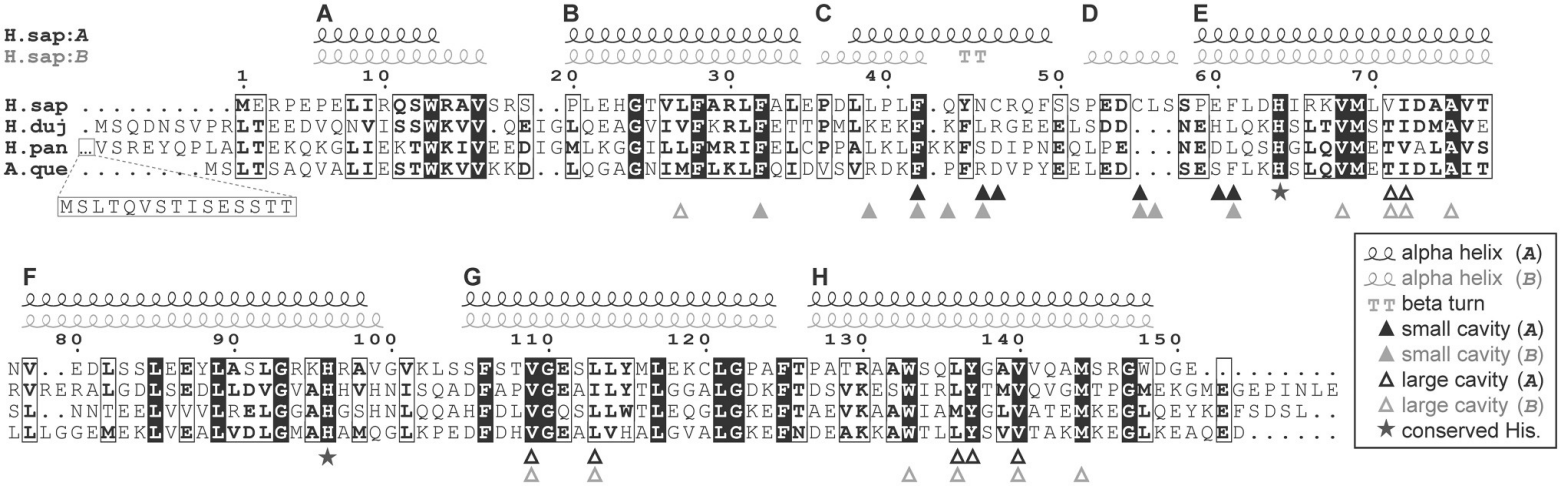
Alignments of NGB protein sequences of *H*. *sapiens* and three sponges. Alignments of NGB protein sequences of *H*. *sapiens* and three sponges: *H*. *dujardini*, *A*. *queenslandica*, and *H*. *panicea*, along with the secondary structure of two monomers (*A* and *B*) of human NGB (PDB ID: 4MPM) made with ESPript web-server [58]. Residues numbering corresponds to human NGB. The helices that form the classical globin fold are labeled from A to H. The human NGB can form a loose homodimer [62]. One of its monomers (type B) maintains a classical eight-helix 3-over-3 alpha-helical sandwich structure (globin fold), whereas another one (type A) lacks the helix D, which is replaced by a coil. Both monomers have two cavities that are thought to regulate the switching between 6- and 5-coordination of the heme, as well as the ligand interaction. Triangles, residues supporting internal cavities; stars, conserved histidines involved in heme coordination; H.sap, *H*. *sapiens* (UniProt ID: Q9NPG2); H.duj, *H*. *dujardini*; A.que, *A*. *queenslandica* (UniProt ID: A0A1X7UGV4); H.pan, *H*. *panicea*.

All principal proteins of the heme biosynthesis pathway were identified in *H*. *dujardini* and *H*. *panicea* transcriptomes. This pathway in sponges includes eight steps similarly to other animals (Fig 3). Seven out of eight heme synthesis factors have medium to strong similarity (60–85%) to analyzed orthologs, while the uroporphyrinogen-III synthase (UROS/hemD) similarity confines to a 50% level. Factors participating in the processing of mature heme have a 40% to 60% similarity. Among them the heme chaperone RSAD1/hemW, which can insert heme into respiratory enzymes and globins, neuroglobin NGB and androglobin ADGB, which are metalloproteins comprising approximately 160–180 and 1300–1500 amino acids respectively. Sequence alignment of NGBs featuring three sponge species along with the secondary structure of human NGB shows solid conservation of important residues supporting globin fold and function, including the proximal and distal histidines (His64 and His96) involved in heme coordination (Fig 4).

The synthesis is initiated in the mitochondria by the transformation of glycine into 5-amino-levulinate (ALA) by 5-aminolevulinate synthase ALAS, then in cytoplasm ALA is converted into porphobilinogen by delta-aminolevulinic acid dehydratase ALAD/hemB and then into hydroxymethylbilane by porphobilinogen deaminase PBGD/hemC. Subsequently, uroporphyrinogen-III synthase UROS/hemD produces uroporphyrinogen III, which is then converted into coproporphyrinogen III by uroporphyrinogen decarboxylase UROD/hemE. Next, coproporphyrinogen III is modified in mitochondria to protoporphyrinogen IX by coproporphyrinogen III oxidase CPOX/HemF. Protoporphyrinogen IX is subsequently transformed into protoporphyrin IX by oxygen-dependent protoporphyrinogen oxidase PPOX/HemY. Finally, protoporphyrin IX is converted to protoheme by the action of ferrochelatase FECH/HemH. The heme chaperone RSAD1/hemW can insert heme into respiratory enzymes. Mature heme may be captured by globins, neuroglobin NGB and androglobin ADGB.

We used a distance-based approach implemented in the Erable software [54] and multiple alignments for 30 eukaryotic species (including three sponges) to obtain the estimates for relative rates of sequence divergence of eight heme biosynthesis enzymes and two globins. The resulted phylogenetic tree is shown in S2 Fig. Following values of the relative rates of sequence divergence were obtained (Fig 2C): ALAD (0.46), FECH (0.52), UROD/hemE (0.56), CPOX/ hemF (0.57), ALAS (0.62), PBGD/hemC (0.71), PPOX/hemY (1.00), UROS/hemD (1.22), ADGB (1.48), NGB (1.87). Among the heme biosynthesis proteins, ALAD is the most evolutionally conserved and UROS/hemD is the fastest evolving. The globins, ADGB and NGB, accumulate amino acid changes more rapidly than the heme synthesis enzymes. NGB shows the highest rate of sequence divergence while maintaining important structural residues conserved (Fig 4). All the data confirm that the heme synthesis pathway is mainly conserved in evolution.

The iron metabolic pathways described in different multicellular organisms specify possible candidates for the iron transport and storage in sponges *H*. *dujardini* and *H*. *panicea* (the accession numbers are in S4 Table). The iron transport protein DMT1 is capable of delivering Fe2+ ions [63,64], whereas SDR2 and NAALAD2 (ancient transferrin receptor homolog) may serve as carriers of Fe3+ ions [65]. The transport protein MFRN (mitoferrin) is a carrier of Fe2+ into mitochondria [66]. The Fe3+ ions are stored in association with FTH1 (ferritin) [67]. The porphyrins, including protoporphyrin IX (PpIX), are transported into mitochondria by the transport protein ABCB6 [68, 69]. The transporter ABCG2 exports PpIX from mitochondria to the cytosol [70–72]. The heme ABCB7 transporter delivers heme to the cytosol [73] and transporter HRG-1 (its homolog identified only in *H*. *dujardini*) delivers it to lysosomes [66]. While HCP1 imports heme into the cell [74], transmembrane protein FPN1 (ferroportin) exports iron from the cell [66]. GST family proteins can also bind heme and may deliver it to mitochondria [75]. The homologs of GST *θ* proteins were found in both sponges, while GST *μ* homolog was only found in *H*. *dujardini*. All enlisted proteins, except for SDR2 and FPN1, have moderate to strong sequence similarity (60% to 80%) to their human and *A*.*queenslandica* orthologs.

Two proteins exemplify so-called moonlighting or multifunctional proteins involved in iron metabolism. The first is glyceraldehyde-3-phosphate dehydrogenase (GAPDH), which was shown to participate in cellular iron balance regulation [76, 77]. The second is iron regulatory protein 1 (IRP1/ACO1) functions as cytoplasmic aconitase, but it can also sense iron concentration and regulate translation by binding to the IREs (iron-responsive elements) in the mRNA of certain genes [78]. The IRP1 homologs were found in both sponges, *H*. *dujardini* and *H*. *panicea*. However, no homologs of the IRP2 paralog were detected. Alignment of IRP1 sequences of three sea sponges and rabbit (*O*. *cuniculus*) revealed conservation of structural motifs and amino acids interacting with nucleotides in respective IREs (S3 Fig). ACO2, the mitochondrial paralog of IRP1/ACO1, has no ascribed function but carries an IRE motif itself. Its homologs were also identified in both sponges. Overall, IRP1/ACO1, ACO2 and GAPDH possess the highest level of homology among analyzed iron metabolism proteins (80–90%).

We also recovered proteins of the PI3K/AKT/mTOR and COX2/AKT/ERK1/2 pathways in *H*. *dujardini* and *H*. *panicea* that are potentially involved in the singlet oxygen damage resistance processes: NFkb1, BCL2, AKT, NOS1, CBS, BIRC5/Survivin, CTH, NAMPT, TST/MPST, CDGSH/mitoNEET, MTOR [12–15]. All these proteins have good sequence similarity (60% to 85%) with the exception of apoptosis regulator BCL2.

Members of the Per/Arnt/Sim transcription factors family linked with iron metabolism and the response to hypoxia were found in both sponges [79]. Namely, there were identified three hypoxia-inducible factors (HIF1α) homologs and one aryl hydrocarbon receptor nuclear translocator (ARNT, also known as HIF1β) homolog. All these factors have a helix-loop-helix and two Per-Arnt-Sim domains (S4–S6 Figs). However, the HIFa/SIM-like homologs in sponges lack the oxygen-dependent degradation domain or the C-terminal transactivation domains present in the human orthologs. Three HIFa/SIM-like sequences in sponges have variable N- and C- termini (S5 Fig). Large sequence variation observed in HIF1A-like and ARNT-like proteins, as well as in SDR2, HRG1 and BCL2 homologs, resulted in unstable alignment coverage and relatively weak overall homology of these proteins (Fig 2A and 2B).

### Expression of the iron metabolism factors during the sponge dissociation and reaggregation processes

**Fig 5:**
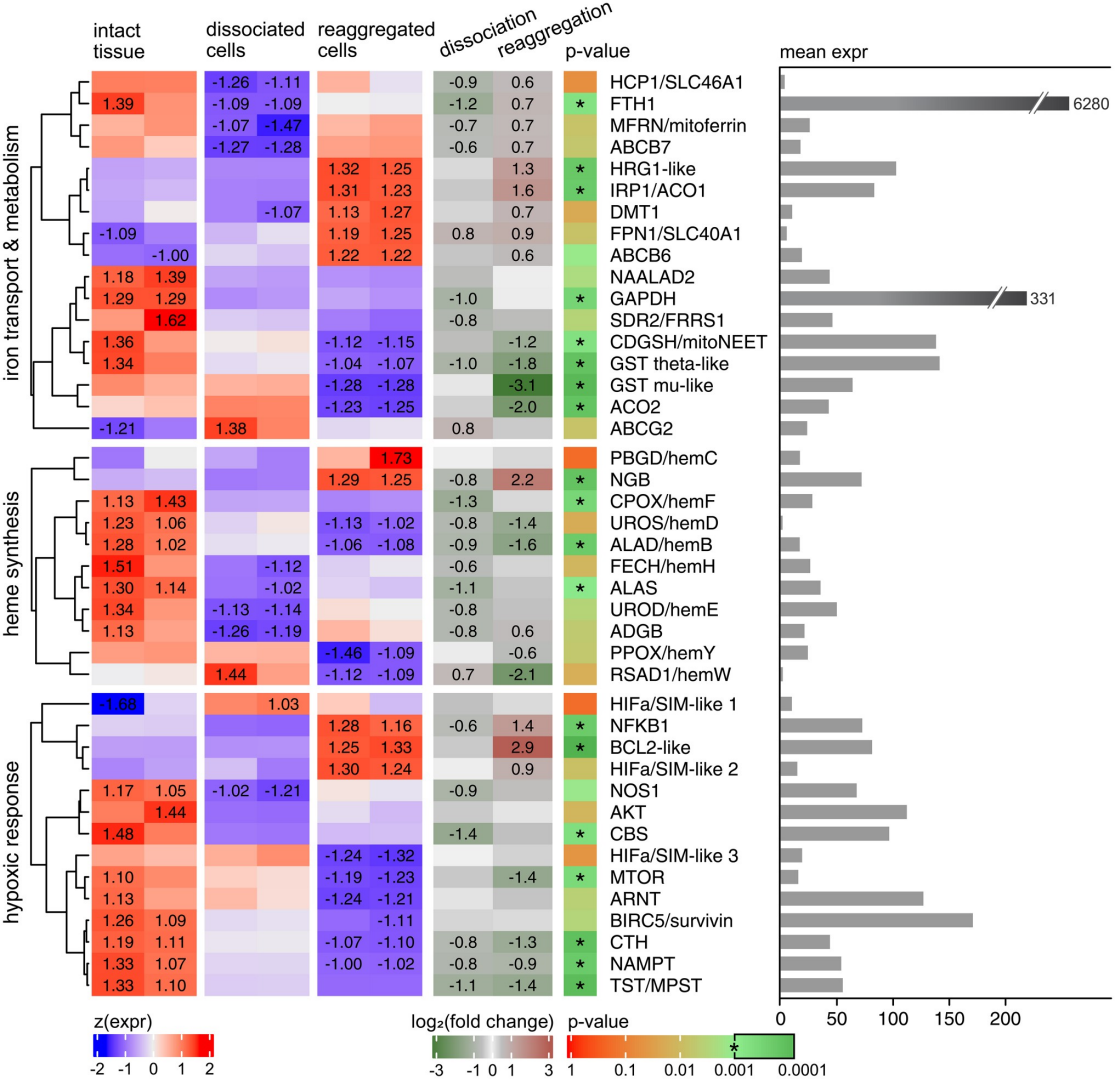
Differential gene expression during the dissociation/reaggregation processes in sponge *H*. *dujardini*. The leftmost heatmap shows z-normalized TMM-corrected CPM expression values computed by edgeR after the removal of low-expressed genes. The second heatmap illustrates log-fold-changes for two state contrasts. The next column shows BH-corrected FDR values, * = FDR < 0.001. The rightmost bar plot demonstrates mean CPM value for all samples.

The regulatory mechanisms operating upon dissociation and reaggregation of sponge cells were studied by RNA-Seq analysis of selected genes in *H*. *dujardini* tissue, dissociated cells and cell aggregates (Fig 5).

Several genes of the iron metabolic pathways changed expression (FDR < 0.001) after the dissociation of the sponge body and then after the reaggregation of dissociated cells (Fig 5). The enzymes of heme biosynthesis (ALAS, ALAD, and others) markedly decreased expression in cells after the body tissue dissociation. Expression of globin ADGB slightly decreased after dissociation and then restored after reaggregation. In contrast, NGB expression was markedly activated after reaggregation (the expression values: 48.22 and 53.74 in tissue, 29.17 and 31.02 in cells, 135.75 and 133.78 in cell aggregates). An increase in expression of HIFA/SIM-like 1, aryl hydrocarbon receptor nuclear translocator ARNT and ABCG2 was observed after body dissociation. Three HIFA/SIM-like homologs tend to have different patterns of expression in each state, however, they did not pass FDR significance threshold. The expression of BCL2 anti-apoptotic protein and NFkB is increased in cell aggregates in comparison to dissociated cell and body tissue samples. CDGSH/mitoNEET, mTOR, TST, and CTH are highly expressed in the body tissue samples, whereas the cell aggregates showed the lowest expression of these proteins. We observed exceptionally high expression of FTH1 in all sponge cell samples with the greatest values in the intact body tissue and the lowest in dissociated cells (9500.14 and 8180.00 in tissues, 3756.40 and 3749.61 in dissociated cells, 6281.02 and 6215.45 after 24h of reaggregation). The metal transporter DMT1 showed higher expression in the cell aggregates than in other cell states. Analysis of the heme transport factors reveals a very high expression of GAPDH in the intact body tissue. The dissociation followed by reaggregation of dissociated cells leads to a significant decrease in the expression of GAPDH and both GST homologs and to an increased expression of HRG1.

**Fig 6:**
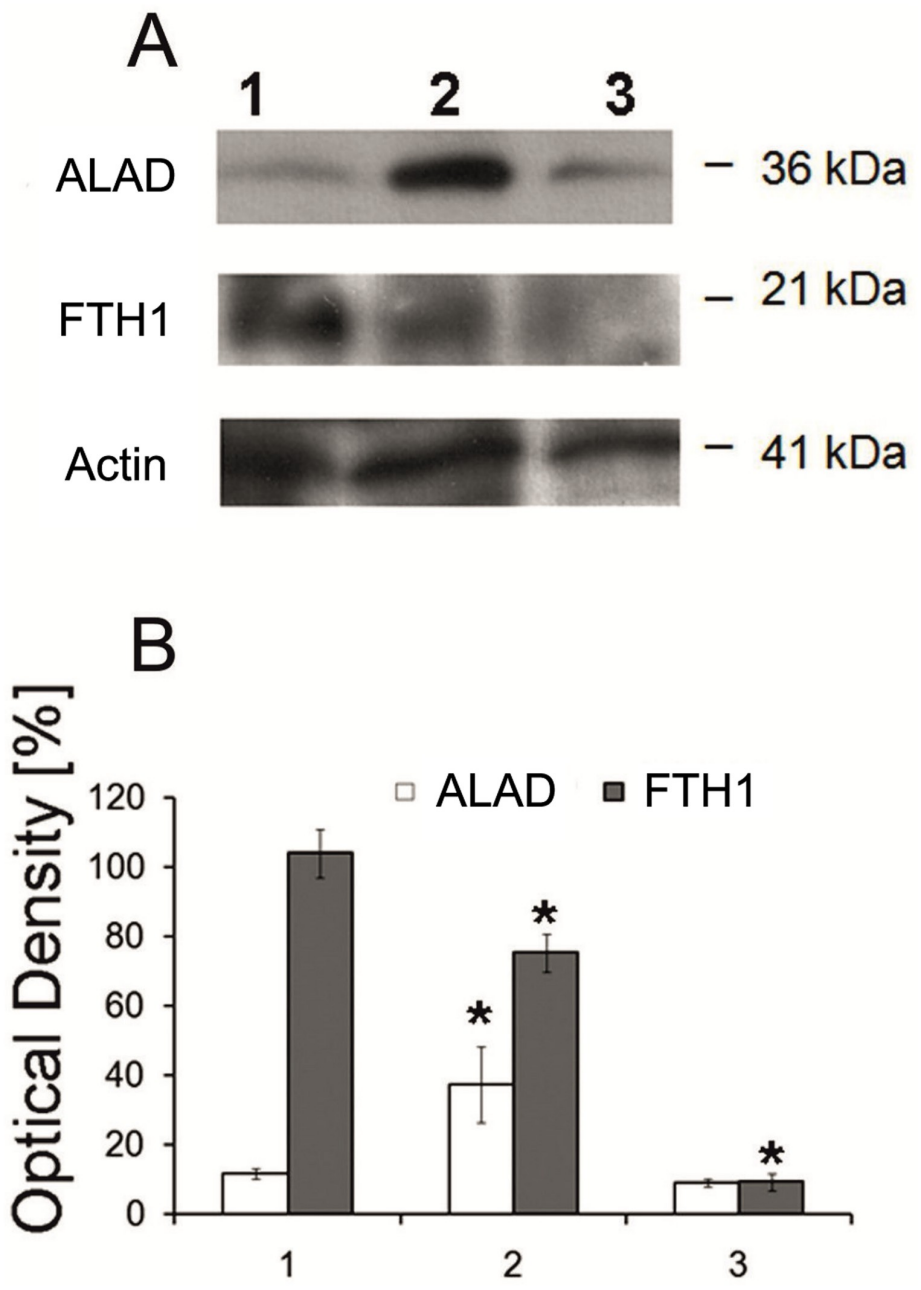
Expression of ALAD and FTH1 proteins in *H*. *dujardini* body tissue, dissociated and aggregated cells. **(A)** Western blot analysis of the proteins in cell extracts performed using antibodies against ALAD and FTH1. **(B)** Relative amounts (represented by the optical density of blots) of ALAD and FTH1 normalized against the amount of β-actin. The amount of FTH1 in tissue was taken as 100%. Mean values ± standard errors are shown. 1) Body tissue; 2) Dissociated cells; 3) Aggregated cells. *Significant difference from control at *p* < 0.05 (*n* ≥ 4).

**Fig 7:**
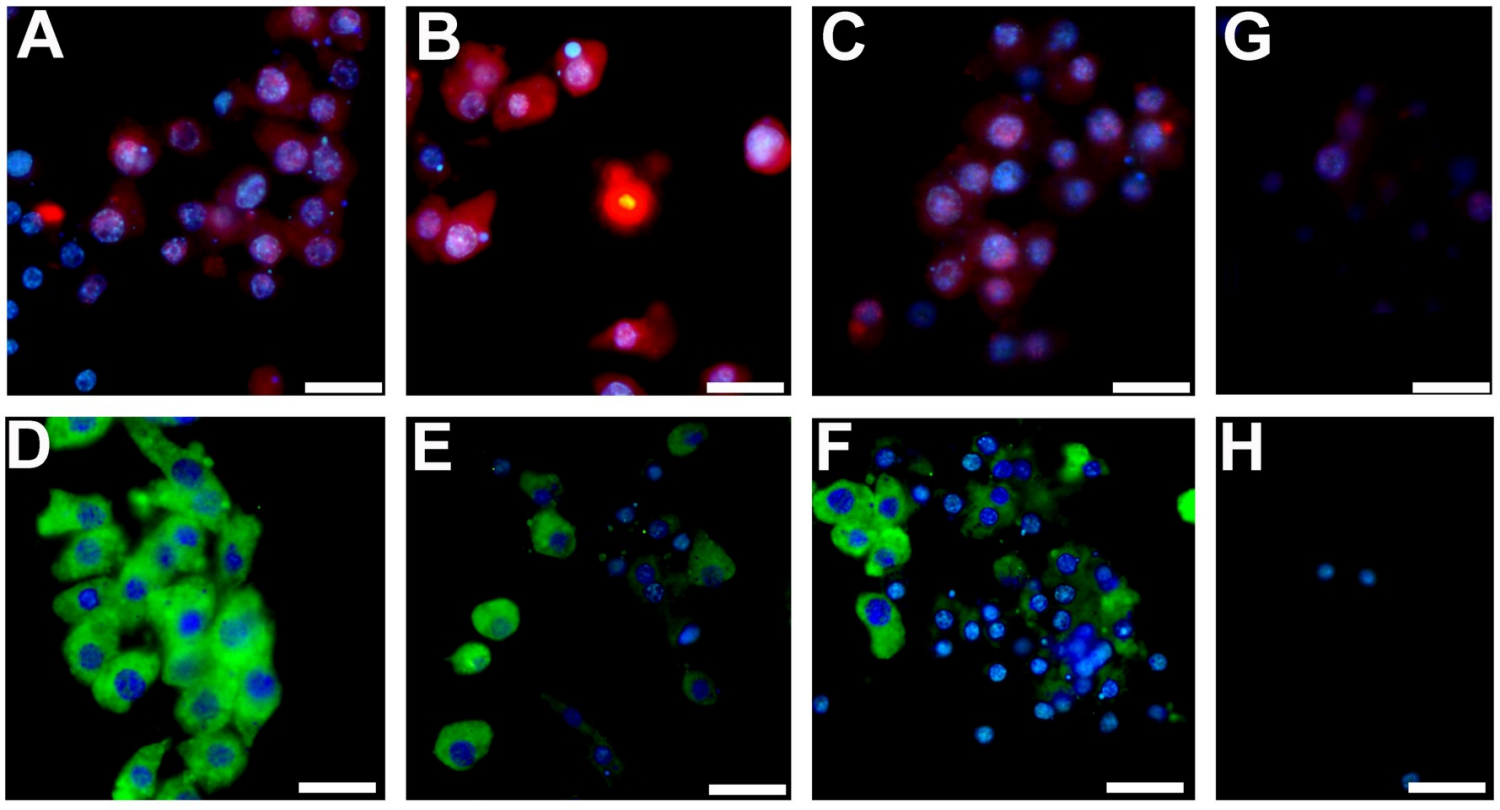
Immune fluorescence of ALAD and FTH1. Immune fluorescence of ALAD (red) and FTH1 (green) in representative samples of *H*. *dujardini* body tissue **(A, D)**, dissociated **(B, E)** and aggregated **(C, F)** cells. Nuclei were visualized by Hoechst-33342 staining (blue). **G, H**–fluorescence control. Scale 10 μm.

In the next experiments, we studied the expression of ALAD and FTH1 in the intact sponge body, dissociated cells, and aggregated cells at a protein level. Both proteins play essential roles in iron metabolism. The enzyme ALAD initiates the heme production in response to oxygen stress in cytoplasm, whereas the iron storage protein ferritin FTH1 controls the labile iron pool. Western blot analysis and immune fluorescence assays showed that ALAD amount in dissociated cells was much higher than in the intact sponge tissue and in the aggregated cells (Figs 6 and 7; S7 Fig). In contrast, the FTH1 amount decreased significantly after the dissociation and was further decreased after the reaggregation of cells (Figs 6 and 7).

**Fig 8:**
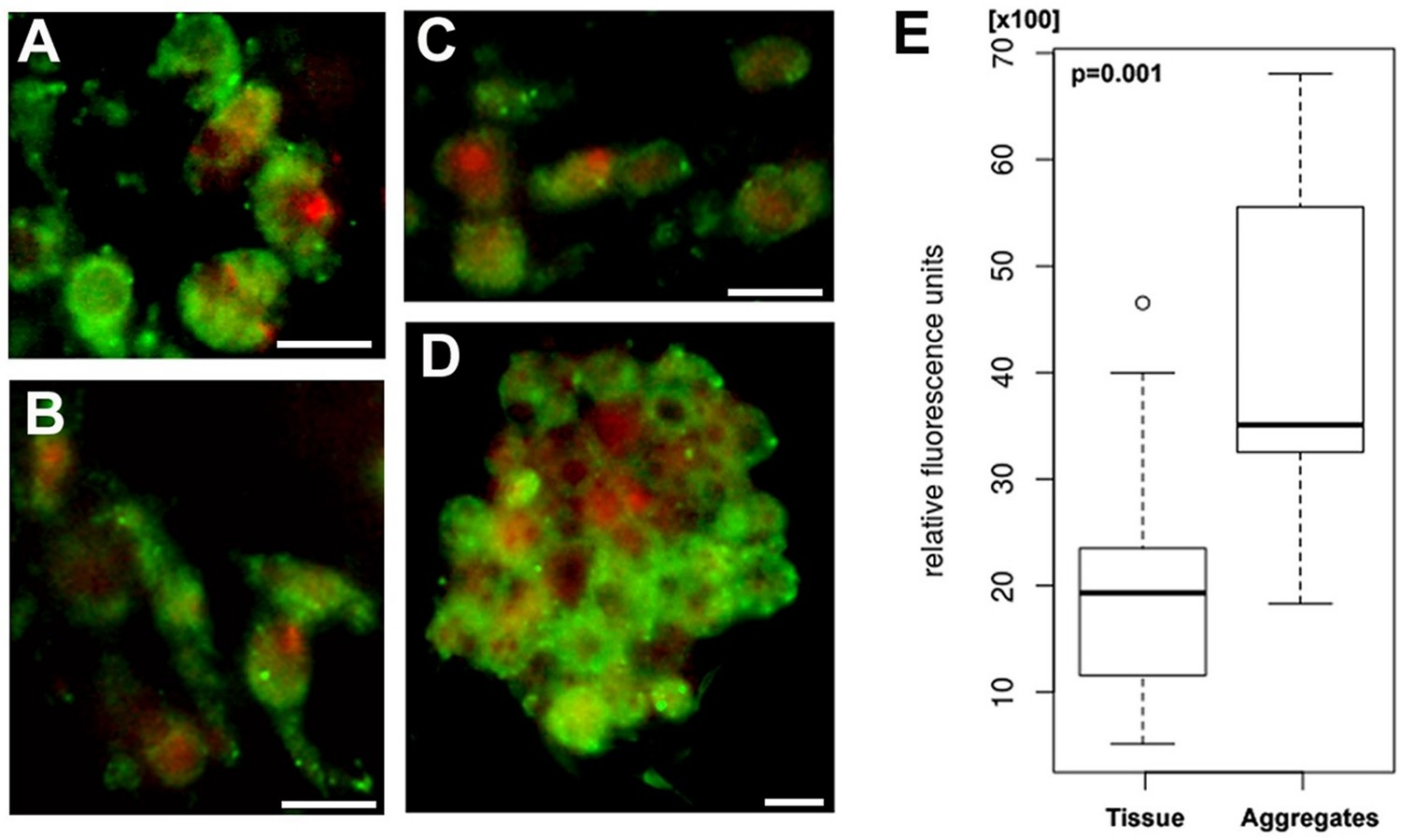
Fluorescence intensity of mitochondria in *H*. *dujardini* body tissues (A, B), dissociated cells (C) and aggregates (D). Mitochondria were visualized with Mitotracker green (green color), nuclei—with DARQ5 fluorochrome (red color). Scale 10 μm. (E) Box-plot for relative fluorescence unit of two group samples (Line, median; box limits, lower, and upper quartiles; whiskers, minimum, and maximum values; (○), type of outliers; p-value for Mann-Whitney’s test).

To further characterize changes in sponges after dissociation/reaggregation processes, we compared the mitochondrial density in the body tissue and aggregated cells. The fluorescence intensity of mitochondria was two times higher in the aggregated cells than in the tissue cells (t = 5.6831, df = 44.194, p = 0.009713) indicating an increase in the mitochondrial density in cells during aggregation (Fig 8).

### Iron regulatory protein (IRP)/iron-responsive element (IRE) pathway in sponges

**Fig 9:**
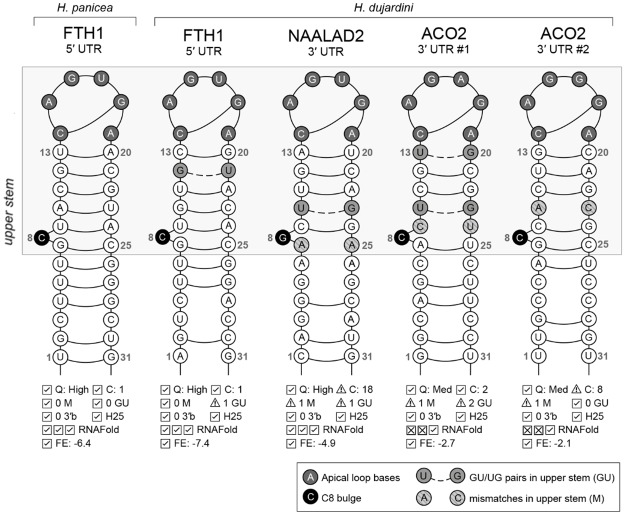
Secondary structures of IREs in FTH1, NAALAD2 and ACO2 mRNAs of *H*. *dujardini* and *H*. *panicea* predicted by SIREs web-server [60]. Q, overall quality determined by SIREs; C, loop class (1, 2: canonical, 3–18: derived from SELEX in silico experiments); M, number of mismatches in upper stem (up to 1 is possible); GU, number of GU/UG pairs in upper stem (up to 2); 3'b, number of bulges at positions 20–23 (up to 1); H25, position 25 is not a G; RNAFold, number of matches with RNAFold predictions: loop structure, upper stem pairings, C8 bulge; FE, free energy.

We found a single IRP homolog in each sponge *H*. *dujardini* and *H*. *panicea* (S4 Table). Its presence suggests a possible involvement of the IRE motifs in the regulation of iron metabolism in Porifera. Binding of IRP1 to IREs located in 5´UTR of mRNAs is reported to inhibit translation, whereas binding to those in 3´UTR stabilizes mRNAs and increases their translation [78]. Search for IREs revealed 25 potential sequences in mRNA of proteins participating in iron pathways in sponges (S5 Table; for mRNA accession numbers see S4 Table). The high-quality IRE motifs were found in the 5´UTR of FTH1 mRNA of both *H*. *dujardini* and *H*. *panicea*, as well as in the 3´UTR of NAALAD2 mRNA of *H*. *dujardini* (Fig 9). Considering that NAALAD2 is an ancient homolog of TFR1(transferrin receptor 1) [65], these findings are consistent with proposed models of IRP/IRE regulation of iron pathway [79, 80]. However, mRNAs of some proteins expected to have IREs (DMT1, FPN1 and MFRN) apparently lack them under this search. The others possess potential IREs in unexpected locations (two motifs of the medium quality were identified in the 3´UTR instead of 5´UTR in ACO2 mRNA of *H*. *dujardini*) (Fig 9, S5 Table). The function of IREs located in the coding regions of mRNAs remains unknown, meanwhile, eight such in-CDS motifs were found, including the high-quality IRE in CTH (cystathionine gamma-lyase) mRNA of *H*. *panicea*. In total, 13 out of 25 motifs were marked as the low-quality IREs. The predicted low quality of respective IREs might be connected to an evolutionary distance from the better investigated mammalian IREs and an imperfect structure of some IREs in sponges.

In contrast to IREs, homologs of IRP1 in sponges show very high sequence similarity to mammalian proteins. Among a total of 902 positions of sequence alignment for three sea sponges and rabbit (*O*. *cuniculus*), 506 amino acids are identical and 149 are functionally similar (S4 Fig). All the IRE-contacting residues, studied in [81], are identical in IRP1s of these four organisms except for two substitutions at positions 379(Lys/Arg) and 685(Asn/Ser). Interestingly, the IRP1 expression triples during the reaggregation of *H*. *dujardini* cells (Fig 5), thus confirming the importance of iron metabolism for morphogenesis in sponges.

## Discussion

**Fig 10:**
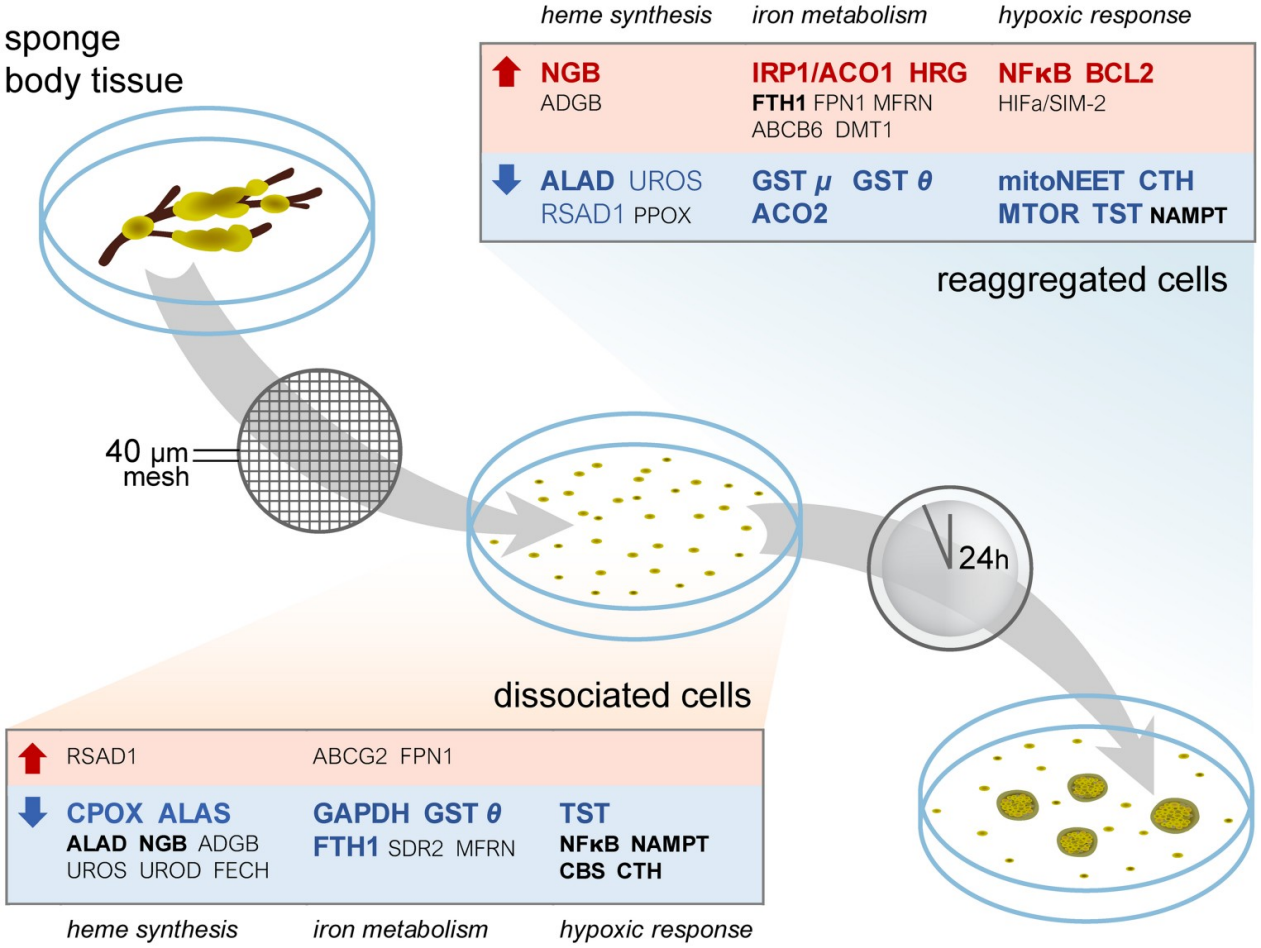
Changes in gene expression during the dissociation and reaggregation processes in *H*. *dujardini*. Genes having FDR < 0.001 are highlighted with bold font. FC, fold change.

Multicellular organisms evolve in oxygenated environments and rely on the respiration for energy generation that is intrinsically connected to iron metabolism [82, 83]. Iron in a form of prosthetic groups is involved in many key cellular processes such as genome replication, oxygen transport and respiration [84–86]. It is not surprising that the sea sponges retain a complete set of factors for iron metabolism and heme synthesis typical for other animals (Fig 3, S4 Table), including members of two ancient globin families, NGB and ADGB [87]. However, our data indicate that sea sponges do not contain an ortholog of globin X (XGB) which was found mostly in fishes, amphibians, and reptiles [88, 89]. The function of XGB is poorly studied and presumably connected to cellular membrane biogenesis [90]. This function may be not essential in sponges or is fulfilled by other proteins. Another scenario is that XGB was lost in sponges and in some other clades. Being ubiquitous, proteins of the iron metabolic pathways have different rates of sequence divergence in evolution. Among the ten proteins involved in heme synthesis, the enzyme ALAD that initiates the heme processing in the cytoplasm is the most conserved, whereas the transporters, globin ADGB and especially globin NGB, evolve at the highest rates (Fig 2C). However, NGB of sponges *H*. *dujardini* and *H*. *panicea* retain approximately 30% identity in amino acid sequences with the human homolog and contain all motifs essential for the protein folding and heme binding. The NGB alignments featuring human and three sponge species show high variability of the N- and C-terminal regions flanking the conserved globin and protoglobin domains. Most of the important residues supporting globin fold and heme coordination are conserved, with the majority of discrepancies present in the C-D region (Fig 4), which is consistent with previous findings [91]. The conservative nature of identified iron metabolic factors corresponds to their important role in animals. However, we did not found in sponges some known proteins of iron metabolism, which are present in more distant classes of invertebrates and mammals [74]: the light chain of ferritin (Ftl), Zip14 (ferrous iron transporter), Dcytb and STEAP2 / 3 (membrane metal reductase transporters), Ceruloplasmin (Cu—binding glycoprotein with ferroxidase activity), Hephaestin (Intestinal and ferroxidase) IREB2 (Regulator of cellular iron homeostasis), mitochondrial transporter ABCB10, Hepcidin / Hemochromatosis / Hemojuvelin (Systemic regulator of iron metabolism and it’s regulators), Tf and Tfr1 / 2 (transferrin and transferrin receptor). The Tfr1 / 2 family is represented in sponges by an ancient homolog, NAALAD2 [65], that has both dimerization (helical) and peptidase domains (S6 Fig). Excess of iron ions is toxic to cells, and the iron concentration is strictly regulated with ferritin FTH1 serving as a cellular depo of iron. The sea-water sponges undergo severe tissue reorganization during their life-cycle, and degenerative and regenerative phenomena are associated with sexual and asexual reproduction [26]. The reaggregation processes begin in the sponge cell suspension immediately after the body dissociation [28], and the morphogenetic potency of dissociated cells depends on iron availability [30]. Our data indicate that the dissociation and reaggregation processes in sponges are associated with dramatic changes in the expression of genes encoding proteins of the iron metabolic pathways (Fig 5). These changes are schematically illustrated in Fig 10.

Oxygenation of sponge cells in the course of body dissociation is accompanied by a decrease in expression of ferritin FTH1 and most genes connected to the heme biosynthesis and hypoxic response. Reaggregation proceeds under an enhanced expression of some factors involved in iron metabolism including globin NGB and hypoxia-inducible factor HIFA/SIM-like 2. Expression of NFκB (p65) and antiapoptotic factor BCL2 was also upregulated in the aggregated cells. Importantly, an over-expression of BCL2, the hypoxia-inducible factor HIFA, inflammation factor NFκB (p65) and neuroglobin NGB characterizes many mammalian malignancies and is regulated in a coordinated manner in the stress response and antiapoptotic pathways [92–96]. The expression of three HIFa homologs in sponges follows different patterns during dissociation/reaggregation processes, and the functional role of each factor remains to be determined. Another problem is that the gene expression data is difficult to interpret in proteomic terms. Translation of mRNA for proteins of iron metabolism in animal cells is under control of the master regulators, iron-regulatory proteins 1 and 2 (IRP1 and IRP2) that sense the iron concentration and regulate mRNA translation by binding to the IRE (iron-responsive element) sequences located usually in the untranslated regions (UTR) of mRNA [78]. The sponge cells contain only a homolog of IRP1. The IRP1 in *H*. *dujardini* presumably participates in positive and negative regulation of mRNA translation. There were 25 IREs identified in mRNAs of sponge proteins involved in iron metabolism (S5 Table). Among them, nine IREs are located in the 5´ UTRs of mRNAs and are likely linked to the inhibition of translation, whereas ten IREs located in the 3´ untranslated regions likely increase the efficiency of translation. Eight IREs were found in the coding regions of mRNAs, the function of such IREs is still unknown. The expression of IRP1 markedly increases under the aggregation of dissociated cells (Fig 5) indicating involvement of IRP1in regulation of morphogenesis in sponges. These results suggest that the IRP-IRE regulation system stems from early metazoans, and its evolutionary development is an exciting biological problem. By using specific antibodies (Figs 6 and 7), we confirmed that cellular concentration of ferritin FTH1 decreases after sponge body dissociation and further decreases after reassociation of cells. This process reflects presumably utilization of stored in ferritin iron ions during dissociation/reaggregation processes. The decay of ferritin might buffer the labile iron pool in sponge cells during morphogenesis. In contrast, the ALAD concentration increases after dissociation and then decreases after reaggregation. The induction of globin NGB in aggregated cells accompanies a marked increase in mitochondrial density (Fig 8), that presumably reflects a high demand for energy in the reaggregation process. All these data indicate a complex mechanism of iron regulation during morphogenesis in sponges that deserves further investigation.

## Conclusions

Sea sponges contain key factors for iron metabolism, the heme biosynthesis and transport. These factors are conserved in evolution although they accumulate amino acid changes with different rates. Among the factors involved in heme processing, the enzyme ALAD is the most conserved, whereas the transport globins, androglobin (ADGB) and neuroglobin (NGB) evolve at the highest rate. The conservative nature of iron metabolic factors is consistent with their important role in Metazoa. The expression of these factors in sponges is under control with the iron-regulatory protein 1/iron-responsive elements (IRP1-IREs) pathway that presumably developed early in evolution. The sea sponges undergo severe tissue reorganization during their life-cycle. The RNA-Seq analysis revealed changes in the expression of iron metabolic factors during the sponge body dissociation followed by cells reaggregation into multicellular aggregates. This result suggests a requirement for regulation of iron metabolism during sponge morphogenesis and indicates an important role of iron metabolic factors in plasticity. The capacity of sponge cells for dedifferentiation and transdifferentiation during morphogenesis resembles the properties of the mammalian stem and cancer cells. Structural plasticity of sea sponges appears to depend on regulation of iron metabolism. The further investigation of sponge cell plasticity and iron metabolism may throw light on general mechanisms of morphogenetic processes in multicellular species.

## Supporting information

S1 Fig Expression correlation heatmap for single-end RNA-seq samples of *H*. *dujardini*. Tiss, intact tissue; cell, cells after dissociation; aggr, aggregates 24h after dissociation. Only genes with the expression of at least 10 CPM in at least one sample were considered.
(PDF)

S2 Fig Phylogenetic tree for 30 eukaryotic species including sponges *H*. *dujardini* and *H*. *panicea* with branch lengths estimated by Erable software [54] using distance matrices for ten proteins involved in heme synthesis. Erable computes branch lengths under the assumption that trees of separate proteins are topologically consistent and using predefined tree topology, which here was inferred from Tree Of Life project. The distance matrix was computed for each protein using its amino acid sequence alignment.
(PDF)

S3 Fig Alignment of IRP1 protein sequences of rabbit *O*. *cuniculus* and three sponges: *H*. *dujardini*, *H*. *panicea*, and *A*. *queenslandica* along with secondary structure of rabbit IRP1 in complex with IRE of FTH1 mRNA (PDB ID: 3SNP) made with ESPript web-server [58]. Residues numbering corresponds to the rabbit IRP1. Triangles, residues contacting with IRE, O.cun, O.cuniculus (UniProt ID: Q01059); H.duj, *H*.*dujardini*; H.pan, *H*.*panicea*; A.que, *A*. *queenslandica* (UniProt ID: A0A1X7VVE4).
(PDF)

S4 Fig HIFa/SIM and ARNT domains alignments with tree clustering for sequences of *H*. *sapiens*, *A*. *queenslandica*, *H*. *dujardini*, *and H*. *panicea*. Domains were predicted using CDVist [55], the trees were constructed with IQ-TREE [56], visualization was made with ete-toolkit [57].
(PDF)

S5 Fig Alignment for HIFa/SIM protein sequences, made and visualized with Jalview2 [59]. (PDF)

S6 Fig Alignment for TFR1 and its homologs along with secondary structure of human TFR1 hd, *H*.*dujardini*; hp, *H*.*panicea*; aqu, *A*. *queenslandica* (NCBI IDs: XP_019859732, XP_019850918); *hsa*, H. sapiens (UniProt IDs: P02786, Q9UP52, Q04609, NP_710163, Q9Y3Q0, Q9UQQ1, NP_996898; PDB ID: 1SUV). (PDF)

S7 Fig Western blots of ALAD, FTH1 and Actin in Halisarca dujardini tissue, dissociated and aggregated cell extracts. Molecular weight markers and proteins are indicated, lanes that did not included in the figure were marked with an “X” above the lane on blot image.
(TIF)

S1 Table Total number of single-end reads per sample for *H*. *dujardini* and their alignment rate reported by RSEM w.r.t. transcriptome assembly made of a separate set of paired-end reads (without decontamination). (PDF)

S2 Table Transrate report for transcriptome assemblies. (PDF)

S3 Table BUSCO report for transcriptome assemblies. (PDF)

S4 Table Accession numbers for protein and mRNA (if it contains iron-responsive element) sequences of genes involved in iron metabolism and the response to hypoxia in *H*. *dujardini* and *H*. *panicea*, and accession numbers to their homologs / best blastp hits in *H*. *sapiens* and *A*. *queenslandica*. (PDF)

S5 Table Features of iron-responsive elements (IREs) in H. dujardini and *H*. *panicea* mRNAs of genes involved in iron metabolism and hypoxic response, predicted by SIREs web-server [60]. (PDF)
