# Correction: Iron metabolic pathways in the processes of sponge plasticity

**DOI:** 10.1371/journal.pone.0230634

**Published:** 2020-03-12

**Authors:** Alexander D. Finoshin, Kim I. Adameyko, Kirill V. Mikhailov, Oksana I. Kravchuk, Anton A. Georgiev, Nicolay G. Gornostaev, Igor A. Kosevich, Victor S. Mikhailov, Guzel R. Gazizova, Elena I. Shagimardanova, Oleg A. Gusev, Yulia V. Lyupina

## Notice of republication

An incorrect version of Fig 10 was published in error. This article was republished on February 28, 2020 to correct for this error. Please download this article again to view the correct version.
