# Supplementary material for: Iron metabolic pathways in the processes of sponge plasticity
Source: PLoS One. 2020 Feb 21;15(2):e0228722. doi: 10.1371/journal.pone.0228722 (PMC7034838; doi:10.1371/journal.pone.0228722)

**S1 Fig. Expression correlation heatmap for single-end RNA-seq samples of *H. dujardini*.**

Tiss, intact tissue; cell, cells after dissociation; aggr, aggregates 24h after dissociation. Only genes with the expression of at least 10 CPM in at least one sample were considered.

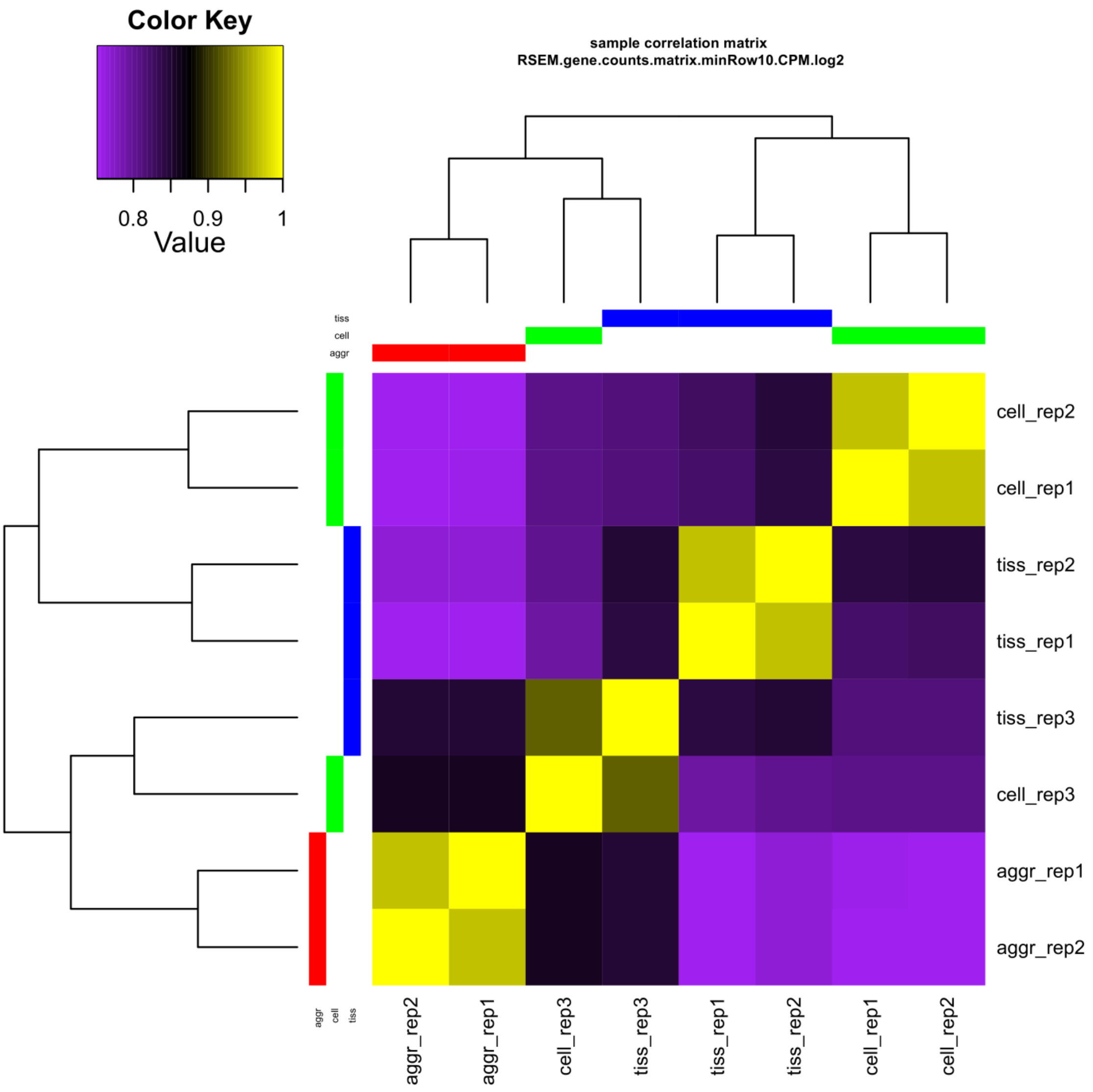

Supplement: S1 Fig — Tiss, intact tissue; cell, cells after dissociation; aggr, aggregates 24h after dissociation. Only genes with the expression of at least 10 CPM in at least one sample were considered. (PDF) [file pone.0228722.s001.pdf]
