# Supplementary material for: Iron metabolic pathways in the processes of sponge plasticity
Source: PLoS One. 2020 Feb 21;15(2):e0228722. doi: 10.1371/journal.pone.0228722 (PMC7034838; doi:10.1371/journal.pone.0228722)

**S2 Fig. Phylogenetic tree for 30 eukaryotic species including sponges *H. dujardini* and *H. panicea* with branch lengths estimated by Erable software** [54] **using distance matrices for ten proteins involved in heme synthesis.** Erable computes branch lengths under the assumption that trees of separate proteins are topologically consistent and using predefined tree topology, which here was inferred from Tree Of Life project. The distance matrix was computed for each protein using its amino acid sequence alignment.

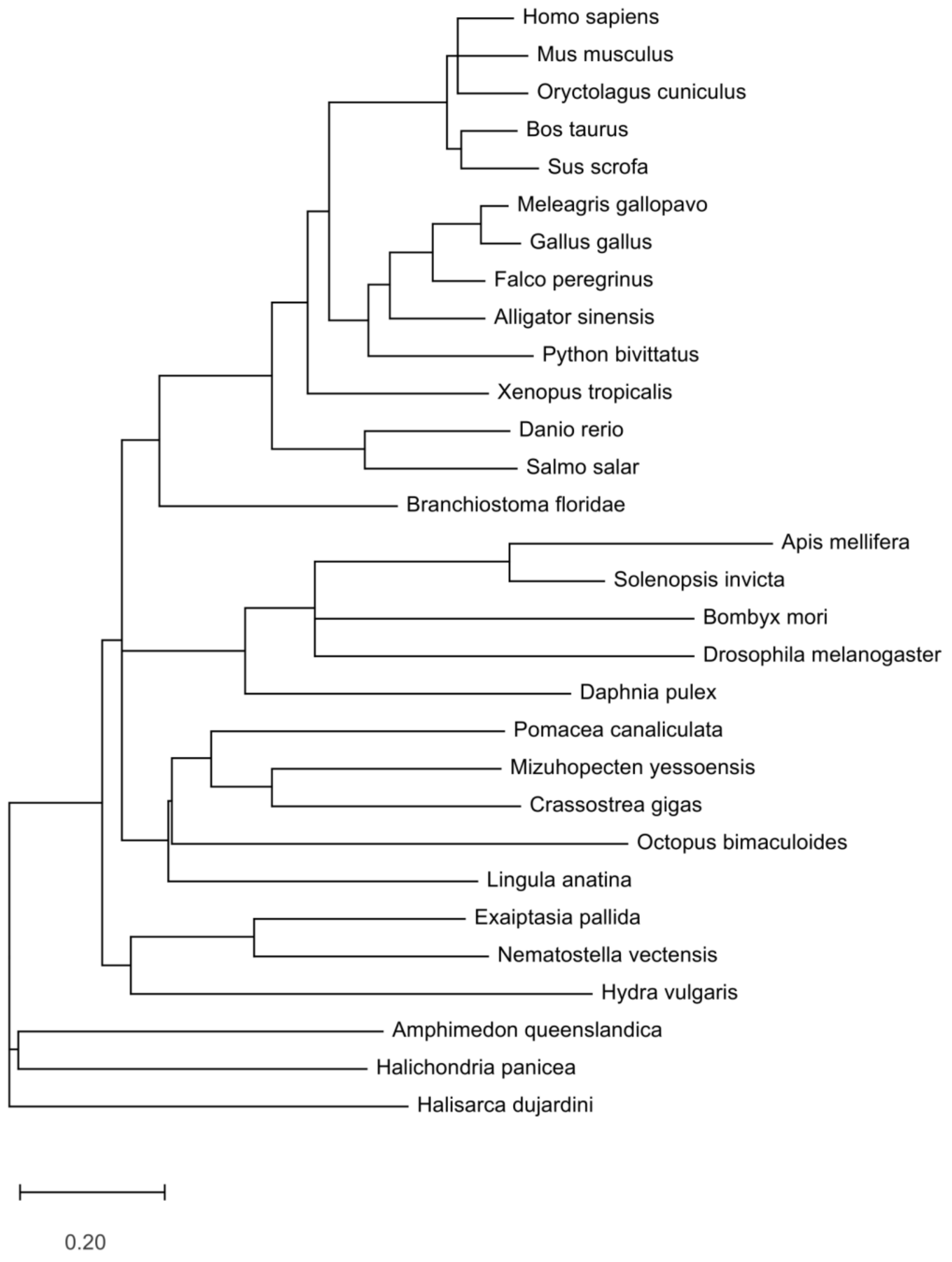

Supplement: S2 Fig — Erable computes branch lengths under the assumption that trees of separate proteins are topologically consistent and using predefined tree topology, which here was inferred from Tree Of Life project. The distance matrix was computed for each protein using its amino acid sequence alignment. (PDF) [file pone.0228722.s002.pdf]
