# Supplementary material for: Iron metabolic pathways in the processes of sponge plasticity
Source: PLoS One. 2020 Feb 21;15(2):e0228722. doi: 10.1371/journal.pone.0228722 (PMC7034838; doi:10.1371/journal.pone.0228722)

S3 Fig. Alignment of IRP1 protein sequences of rabbit *O. cuniculus* and three sponges: *H. dujardini*, *H. panicea*, and *A. queenslandica* along with secondary structure of rabbit IRP1 in complex with IRE of FTH1 mRNA (PDB ID: 3SNP) made with ESPript web-server [58]**.** Residues numbering corresponds to the rabbit IRP1. Triangles, residues contacting with IRE, O.cun, O.cuniculus (UniProt ID: Q01059); H.duj, *H.dujardini*; H.pan, *H.panicea*; A.que, *A. queenslandica* (UniProt ID: A0A1X7VVE4).

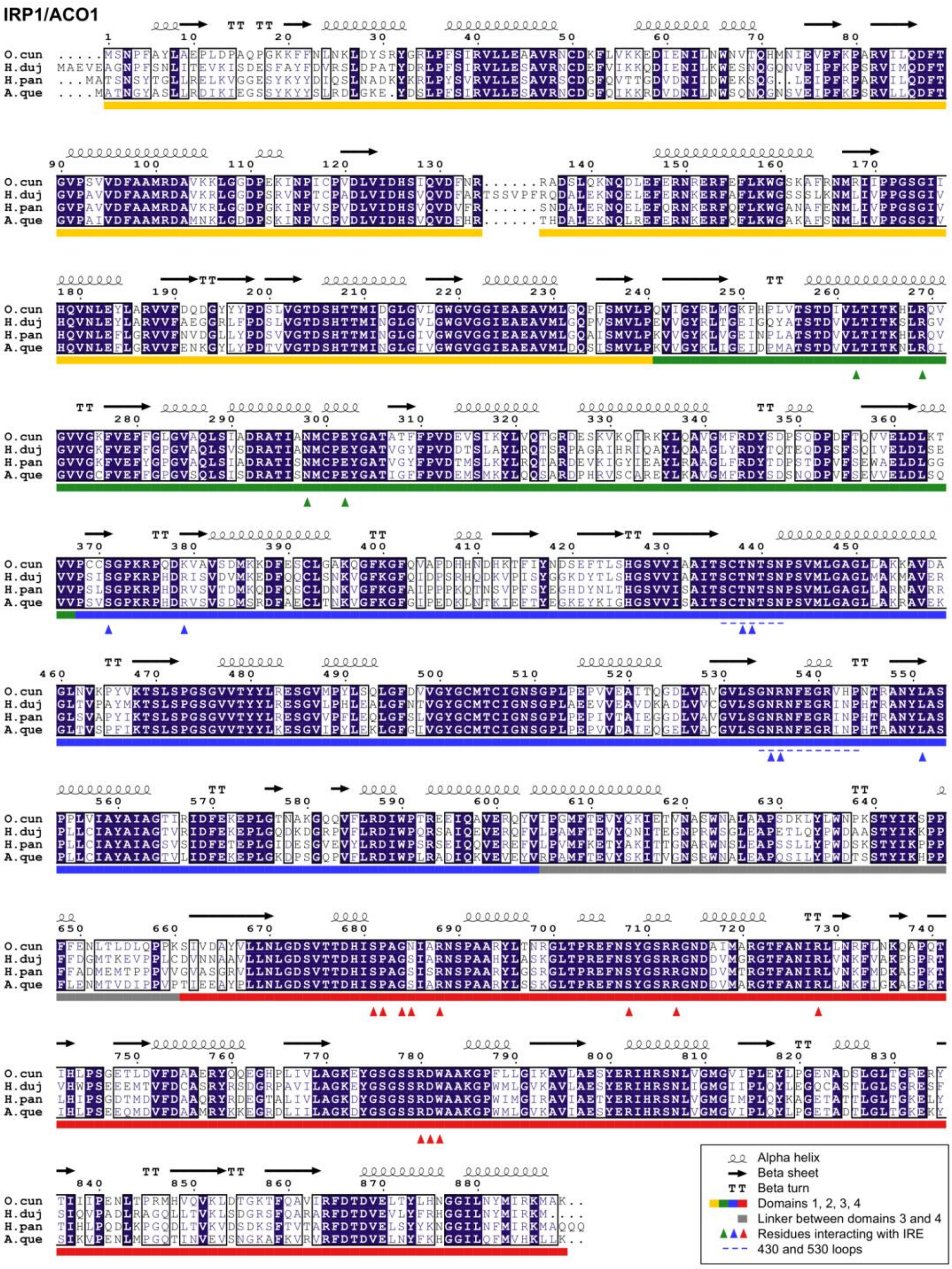

Supplement: S3 Fig — Residues numbering corresponds to the rabbit IRP1. Triangles, residues contacting with IRE, O.cun, O.cuniculus (UniProt ID: Q01059); H.duj, H.dujardini; H.pan, H.panicea; A.que, A. queenslandica (UniProt ID: A0A1X7VVE4). (PDF) [file pone.0228722.s003.pdf]
