# Supplementary material for: Iron metabolic pathways in the processes of sponge plasticity
Source: PLoS One. 2020 Feb 21;15(2):e0228722. doi: 10.1371/journal.pone.0228722 (PMC7034838; doi:10.1371/journal.pone.0228722)

S4 Fig. HIFa/SIM and ARNT domains alignments with tree clustering for sequences of *H. sapiens*, *A. queenslandica*, *H. dujardini*, and *H. panicea*. Domains were predicted using CDVist [55], the trees were constructed with IQ-TREE [56], visualization was made with ete-toolkit [57].

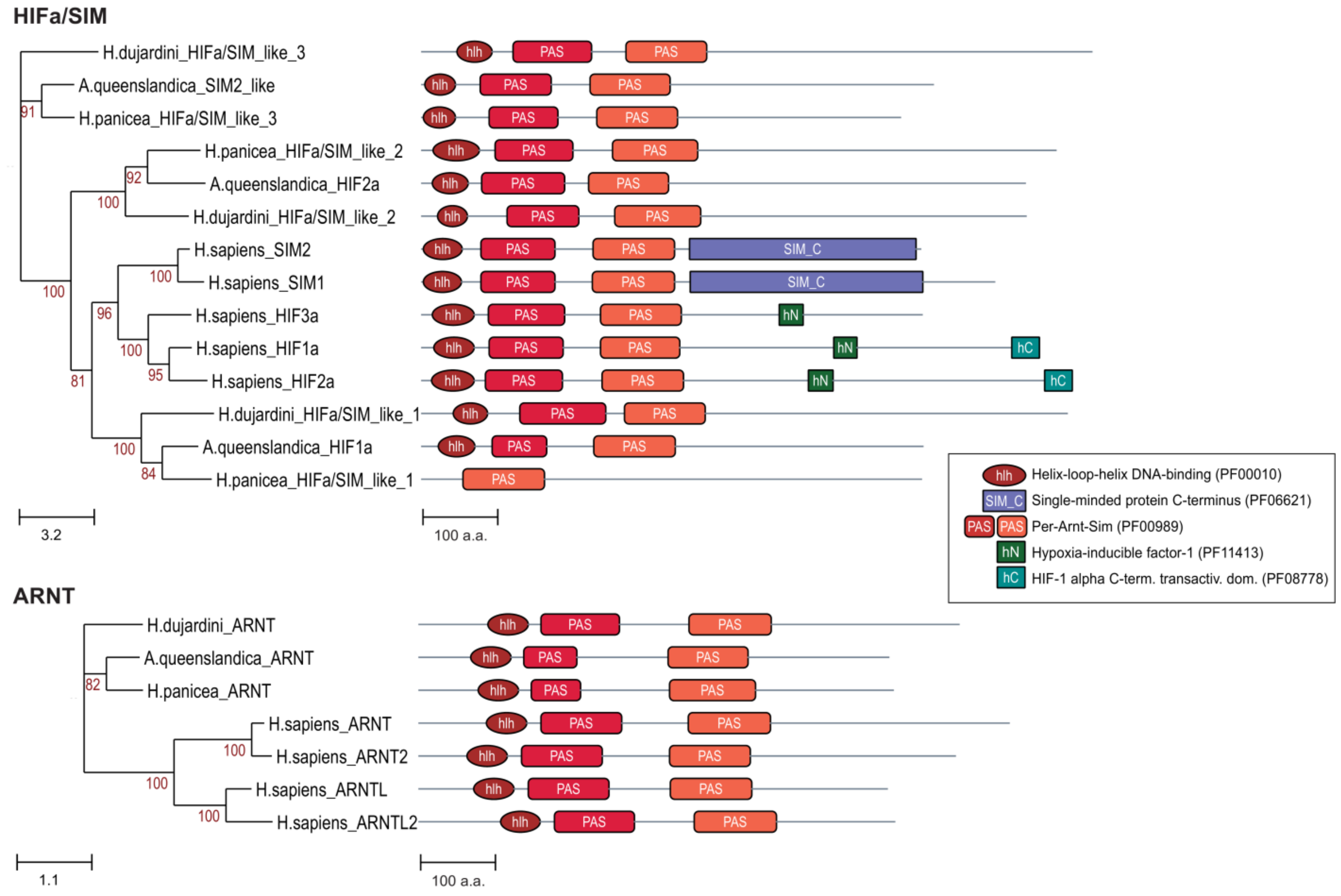

Supplement: S4 Fig — Domains were predicted using CDVist [55], the trees were constructed with IQ-TREE [56], visualization was made with ete-toolkit [57]. (PDF) [file pone.0228722.s004.pdf]
