# Supplementary figures and images for: Iron metabolic pathways in the processes of sponge plasticity
Source: PLoS One. 2020 Feb 21;15(2):e0228722. doi: 10.1371/journal.pone.0228722 (PMC7034838; doi:10.1371/journal.pone.0228722)

[59].

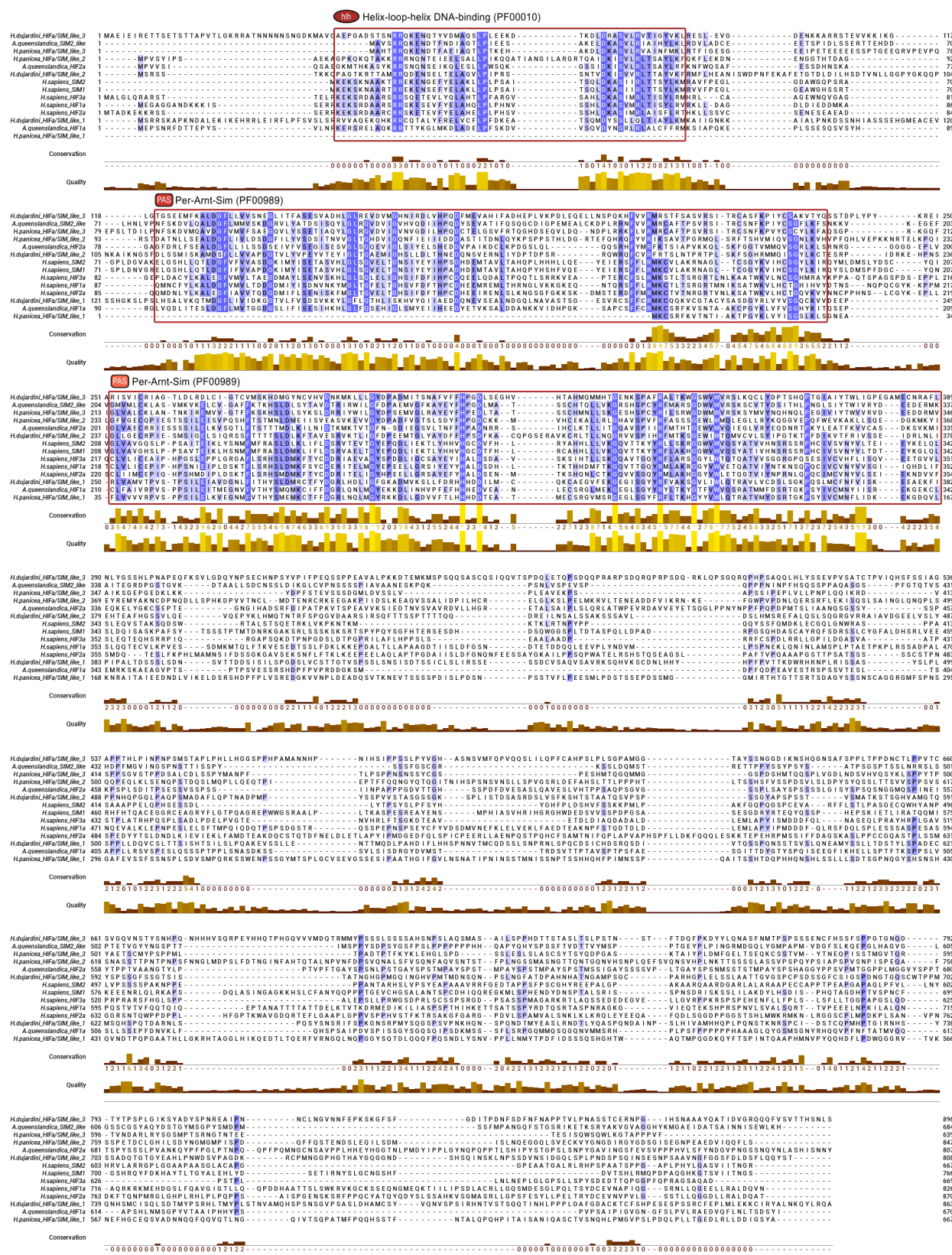

Supplement: S5 Fig — (PDF) [file pone.0228722.s005.pdf]

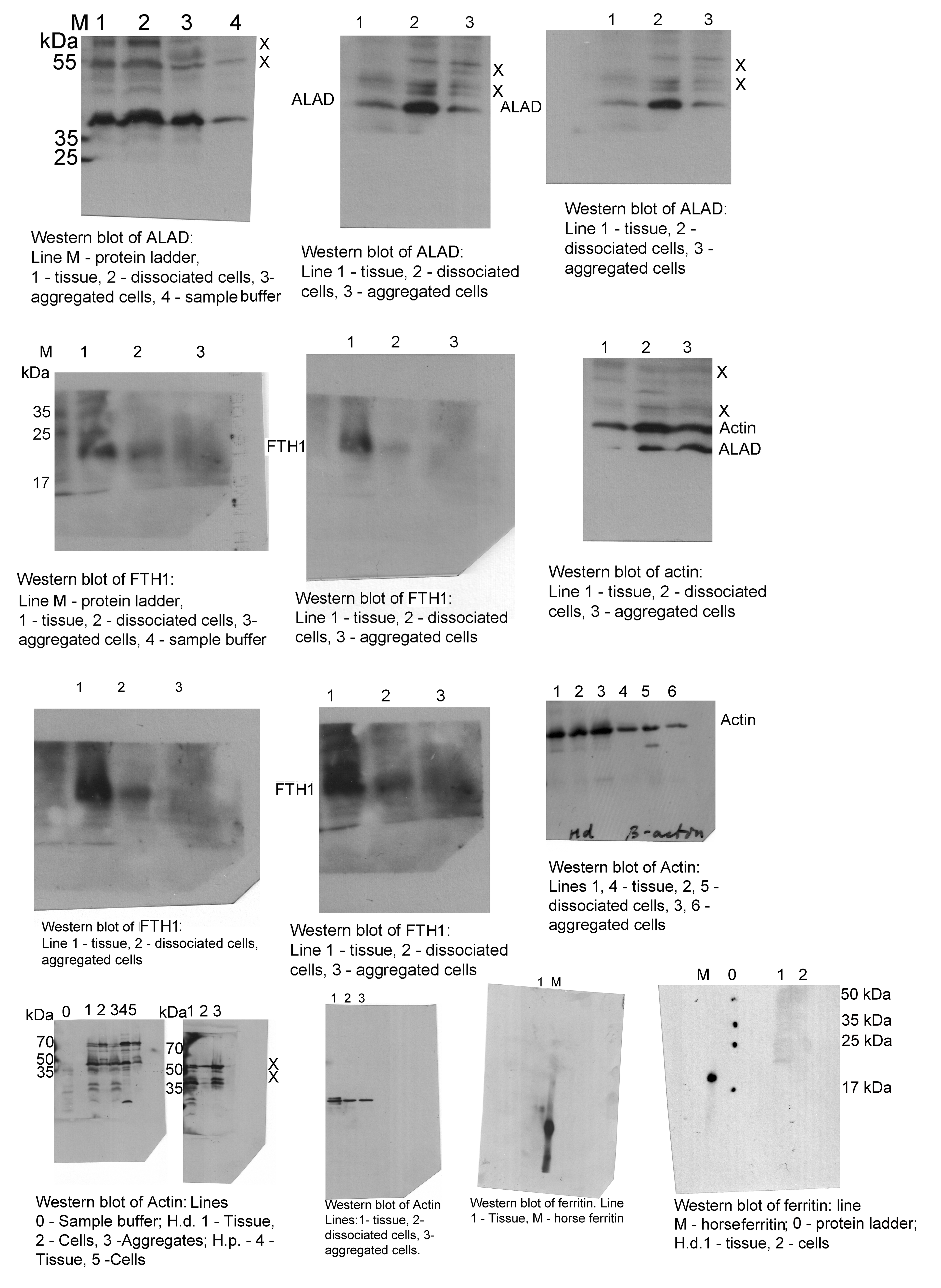

Supplement: S7 Fig — Molecular weight markers and proteins are indicated, lanes that did not included in the figure were marked with an “X” above the lane on blot image. (TIF) [file pone.0228722.s007.tif]
