# Supplementary material for: Iron metabolic pathways in the processes of sponge plasticity
Source: PLoS One. 2020 Feb 21;15(2):e0228722. doi: 10.1371/journal.pone.0228722 (PMC7034838; doi:10.1371/journal.pone.0228722)
Supplement: S6 Fig — (PDF) [file pone.0228722.s006.pdf]

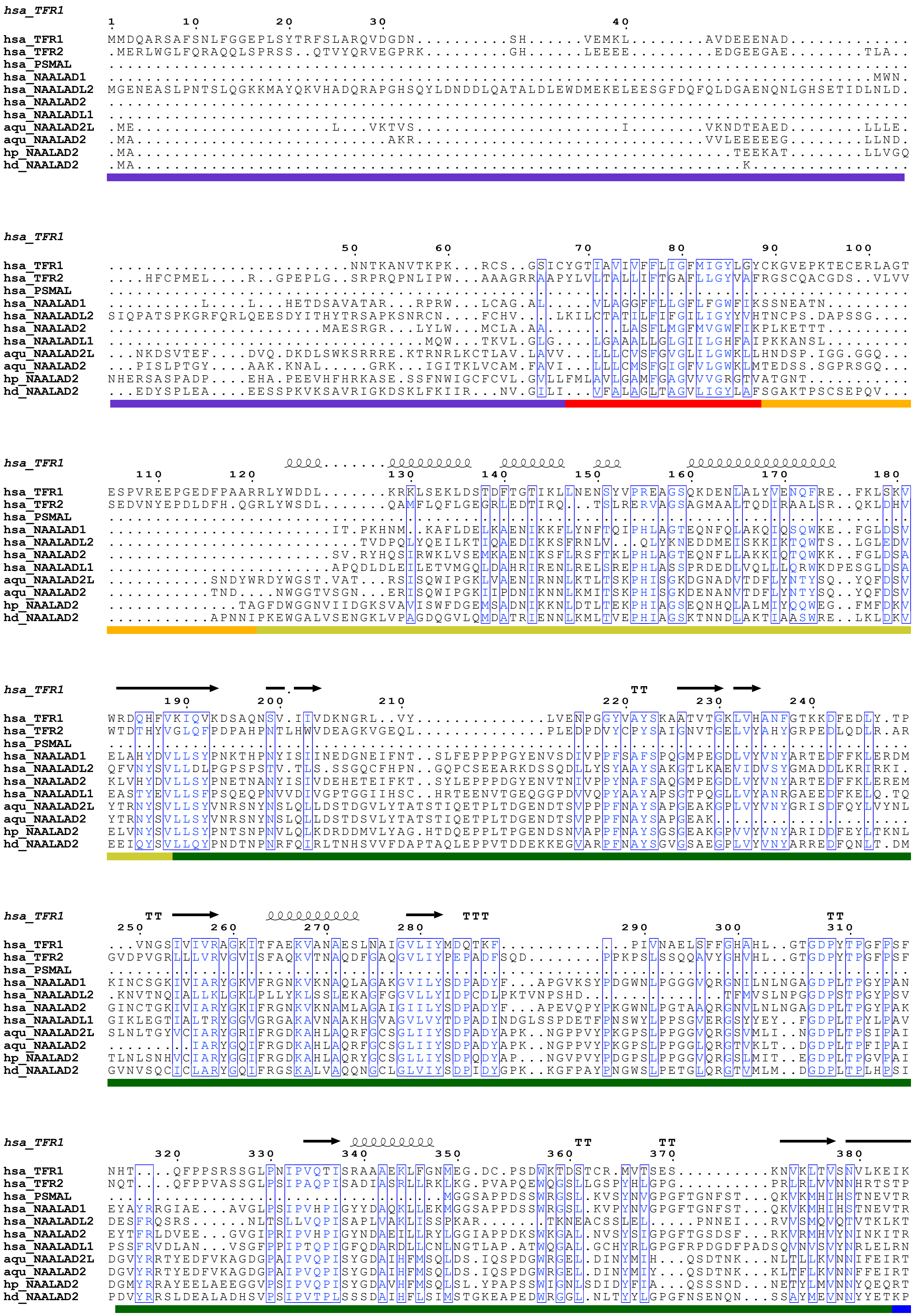
hsa_TFR1
1 10 20 30 40
hsa_TFR1 MMDQARSAFSNLFGGEPLSYTRFSLARQVDGDN..........SH......VEMKL........AVDEEENAD.............
hsa_TFR2 .MERLWGLFQRAQQLSPRSS..QTVYQRVEGPRK.........GH......LEEEE.........EDGEEGAE........TLA..
hsa_PSMAL ....................................................................................
hsa_NAALAD1 .................................................................................MWN.
hsa_NAALADL2 MGENEASLPNTSLQGKKMAYQKVHADQRAPGHSQYLDNDDLQATALDLEWDMEKELEESGFDQFQLDGAENQNLGHSETIDLNLD.
hsa_NAALAD2 ....................................................................................
hsa_NAALADL1 ....................................................................................
aqu_NAALAD2L .ME..................L...VKTVS...................I.......VKNDTEAED........LLLE.
aqu_NAALAD2 .MA...................AKR.........................VVLEEEEEG........LLND.
hp_NAALAD2 .MA.........................................................TEEKAT........LLVGQ
hd_NAALAD2 .MA...........................................................K..................
hsa_TFR1
50 60 70 80 90 100
hsa_TFR1 .........................NNTKANVTKPK...RCS..GSICYGTIAVIVFFLIGFMIGYLGYCKGVEPKTECERLAGT
hsa_TFR2 ....HFCPMEL....R..GPEPLG..SRPRQPNLIPW...AAAGRRAAPYLVLTALLIFTGAFLLGYVAFRGSCQACGDS..VLVV
hsa_PSMAL ....................................................................................
hsa_NAALAD1 ..........L....L...HETDSAVATAR...RPRW...LCAG.AL.....VLAGGFFLLGFLFGWFIKSSNEATN.........
hsa_NAALADL2 SIQPATSPKGRFQRLQEESDYITHYTRSAPKSNRCN....FCHV....LKILCTATILFIFGILIGYYVHTNCPS.DAPSSG....
hsa_NAALAD2 ........................MAESRGR...LYLW...MCLA.AA........LASFLMGFMVGWFIKPLKETTT.........
hsa_NAALADL1 ..................................MQW...TKVL.GLG....LGAAALLGLGIILGHFAIPKKANSL.........
aqu_NAALAD2L ...NKDSVTEF....DVQ.DKDLSWKSRRRE.KTRNRLKCTLAV.LAVV...LLLCVSFGVGLILGWKLLHNDSP.IGG.GGQ...
aqu_NAALAD2 ...PISLPTGY....AAK.KNAL.....GRK...IGITKLVCAM.FAVI...LLLCMSFGIGFVLGWKLMTEDSS.SGPRSGQ...
hp_NAALAD2 NHERSASPADP....EHA.PEEVHFHRKASE.SSFNWIGCFCVL.GVLLFMLAVLGAMFGAGVVVGRGTVATGNT..........
hd_NAALAD2 ...EDYSPLEA....EESSPKVKSAVRIGKDSKLFKIIR...NV.GILI...VFALAGLTAGVLIGYLAFSGAKTPSCSEPQV...
hsa_TFR1
110 120 130 140 150 160 170 180
hsa_TFR1 ESPVREEPGEDFPAARRLYWDDL.......KRKLSEKLDSTDFTGTIKLLNENSYVPREAGSQKDENLALYVENQFRE..FKLSKV
hsa_TFR2 SEDVNYEPDLDFH.QGRLYWSDL.......QAMFLQFLGEGRLEDTIRQ...TSLRERVAGSAGMAALTQDIRAALSR..QKLDHV
hsa_PSMAL ....................................................................................
hsa_NAALAD1 ........................IT.PKHNM..KAFLDELKAENIKKFLYNFTQIPHLAGTEQNFQLAKQIQSQWKE..FGLDSV
hsa_NAALADL2 ...........................TVDPQLYQEILKTIQAEDIKKSFRNLV...QLYKNEDDMEISKKIKTQWTS..LGLEDV
hsa_NAALAD2 ........................SV.RYHQSIRWKLVSEMKAENIKSFLRSFTKLPHLAGTEQNFLLAKKIQTQWKK..FGLDSA
hsa_NAALADL1 ........................APQDLDLEILETVMGQLDAHRIRENLRELSREPHLASSPRDEDLVQLLLQRWKDPESGLDSA
aqu_NAALAD2L ...........SNDYWRDYWGST.VAT...RSISQWIPGKLVAENIRNNLKTLTSKPHISGKDGNADVTDFLYNTYSQ..YQFDSV
aqu_NAALAD2 ...........TND....NWGGTVSGN...ERISQWIPGKIIPDNIKNNLKMITSKPHISGKDENANVTDFLYNTYSQ..YQFDSV
hp_NAALAD2 ..............TAGFDWGGNVIIDGKSVAVISWFDGEMSADNIKKNLDTLTEKPHIAGSEQNHQLALMIYQQWEG..FMFDKV
hd_NAALAD2 ...........APNNIPKEWGALVSENGKLVPAGDQGVLQMDATRIENNLKMLTVEPHIAGSKTNNDLAKTIAASWRE..LKLDKV
hsa_TFR1
TT
190 200 210 220 230 240
hsa_TFR1 WRDQHFVKIQVKDSAQNSV.IIVDKNGRL..VY.............LVENPGGYVAYSKAATVTGKLVHANFGTKKDFEDLY.TP
hsa_TFR2 WTDTHYVGLQFPDPAHPNTLHWVDEAGKVGEQL.............PLEDPDVYCPYSAIGNVTGELVYAHYGRPEDLQDLR.AR
hsa_PSMAL ....................................................................................
hsa_NAALAD1 ELAHYDVLLSYPNKTHPNYISIINEDGNEIFNT..SLFEPPPPGYENVSDIVPPFSAFSPQGMPEGDLVYVNYARTEDFFKLERDM
hsa_NAALADL2 QFVNYSVLLDLPGPSPSTV.TLS.SSGQCFHPN..GQPCSEEARKDSSQDLLYSYAAYSAKGTLKAEVIDVSYGMADDLKRIRKI.
hsa_NAALAD2 KLVHYDVLLSYPNETNANYISIVDEHETEIFKT..SYLEPPPDGYENVTNIVPPYNAFSAQGMPEGDLVYVNYARTEDFFKLEREM
hsa_NAALADL1 EASTYEVLLSFPSQEQPNVVDIVGPTGGIIHSC..HRTEENVTGEQGGPDVVQPYAAYAPSGTPQGLLVYANRGAEEDFKELQ.TQ
aqu_NAALAD2L YTRNYSVLLSYVNRSNYNSLQLLDSTDGVLYTATSTIQETPLTDGENDTSVPPPFNAYSAPGEAKGPLVYVNYGRISDFQYLVYNL
aqu_NAALAD2 YTRNYSVLLSYVNRSNYNSLQLLDSTDSVLYTATSTIQETPLTDGENDTSVPPPFNAYSAPGEAK....................
hp_NAALAD2 ELVNYSVLLSYPNTSNPNVLQLKDRDDMVLYAG.HTDQEPPLTPGENDSNVAPPFNAYSGSGEAKGPVVYVNYARIDDFEYLTKNL
hd_NAALAD2 EEIQYSVLLQYPNDTNPNRFQIRLTNHSVVFDAPTAQLEPPVTDDEKKEGVARPFNAYSGVGSAEGPLVYVNYARREDFQNLT.DM
hsa_TFR1
TT TTT TT
250 260 270 280 290 300 310
hsa_TFR1 ...VNGSIVIVRAGKITFAEKVANAESLNAIGVLIYMDQTKF...........PIVNAELSFFGHAHL..GTGDPYTPGFPSF
hsa_TFR2 GVDPVGRLLLVRVGVISFAQKVTNAQDFGAQGVLIYPEPADFSQD........PPKPSLSSQQAVYGHVHL..GTGDPYTPGFPSF
hsa_PSMAL ....................................................................................
hsa_NAALAD1 KINCSGKIVIARYGKVFRGNKVKNAQLAGAKGVILYSDPADYF...APGVKSYPDGWNLPGGGVQRGNILNLNGAGDPLTPGYPAN
hsa_NAALADL2 .KNVTNQIALLKLGKLPLLYKLSSLEKAGFGGVLLYIDPCDLPKTVNPSHD...............TFMVSLNPGGDPSTPGYPSV
hsa_NAALAD2 GINCTGKIVIARYGKIFRGNKVKNAMLAGAIGIILYSDPADYF...APEVQPYPKGWNLPGTAAQRGNVLNLNGAGDPLTPGYPAK
hsa_NAALADL1 GIKLEGTIALTRYGGVGRGAKAVNAAKHGVAGVLVYTDPADINDGLSSPDETFPNSWYLPPSGVERGSYYEY..FGDPLTPYLPAV
aqu_NAALAD2L SLNLTGYVCIARYGRIFRGDKAHLAQRFGCSGLIIYSDPADYAPK..NGPPVYPKGPSLPPGGVQRGSVMLI..NGDPLTPSIPAI
aqu_NAALAD2 ..........IARYGQIFRGDKAHLAQRFGCSGLIIYSDPADYAPK..NGPPVYPKGPSLPPGGLQRGTVKLT..DGDPLTPFIPAI
hp_NAALAD2 TLNLSNHVCIARYGGIFRGDKAHLAQRYGCSGLLIYSDPQDYAP...NGVPVYPDGPSLPPGGVQRGSLMIT..EGDPLTPGVPAI
hd_NAALAD2 GVNVSQCICLARYGQIFRGSKALVAQQNGCLGLVIYSDPIDYGPK..KGFPAYPNGWSLPETGLQRGTVMLM..DGDPLTPLHPSI
hsa_TFR1
TT TT
320 330 340 350 360 370 380
hsa_TFR1 NHT....QFPPSRSSGLPNIPVQTISRAAAEKLFGNMEG.DC.PSDWKTDSTCR.MVTSES..........KNVKLTVSNVLKEIK
hsa_TFR2 NQT....QFPPVASSGLPSIPAQPISADIASRLLRKLKG.PVAPQEWQGSLLGSPYHLGPG..........PRLRLVVNNHRTSTP
hsa_PSMAL ................................MGGSAPPDSSWRGSL.KVSYNVGPGFTGNFST...QKVKMHIHSTNEVTR
hsa_NAALAD1 EYAYRRGIAE...AVGLPSIPVHPIGYYDAQKLLEKMGGSAPPDSSWRGSL.KVPYNVGPGFTGNFST...QKVKMHIHSTNEVTR
hsa_NAALADL2 DESFRQSRS......NLTSLLVQPISAPLVAKLISSPKAR.......TKNEACSSLEL.....PNNEI...RVVSMQVQTVTKLKT
hsa_NAALAD2 EYTFRLDVEE...GVGIPRIPVHPIGYNDAEILLRYLGGIAPPDKSWKGAL.NVSYSIGPGFTGSDSF...RKVRMHVYNINKITR
hsa_NAALADL1 PSSFRVDLAN...VSGFPPIPTQPIGFQDARDLLCNLNGTLAP.ATWQGAL.GCHYRLGPGFRPDGDFPADSQVNVSVYNRLELRN
aqu_NAALAD2L DGVYRRTYEDFVKAGDGPAIPVQPISYGDAIHFMSQLDS.IQSPDGWRGEL.DINYMIH...QSDTNK...NLTLLKVNNIFEIRT
aqu_NAALAD2 DGVYRRTYEDFVKAGDGPAIPVQPISYGDAIHFMSQLDS.IQSPDGWRGEL.DINYMIY...QSDTNK...KLTFLKVNNFFEIRT
hp_NAALAD2 DGMYRRAYEELAEEGGVPSIPVQPISYGDAVHFMSQLSL.YPAPSSWIGNLSDIDYFIA...QSSSND...NETYLMVNNYQEQRT
hd_NAALAD2 PDVYRRSLDEALADHSVPSIPVTPLSSSDAIHFLSIMSTGKEAPEDWRGGL.NLTYYLGPGFNSENQN...SSAYMEVNNYYETKP

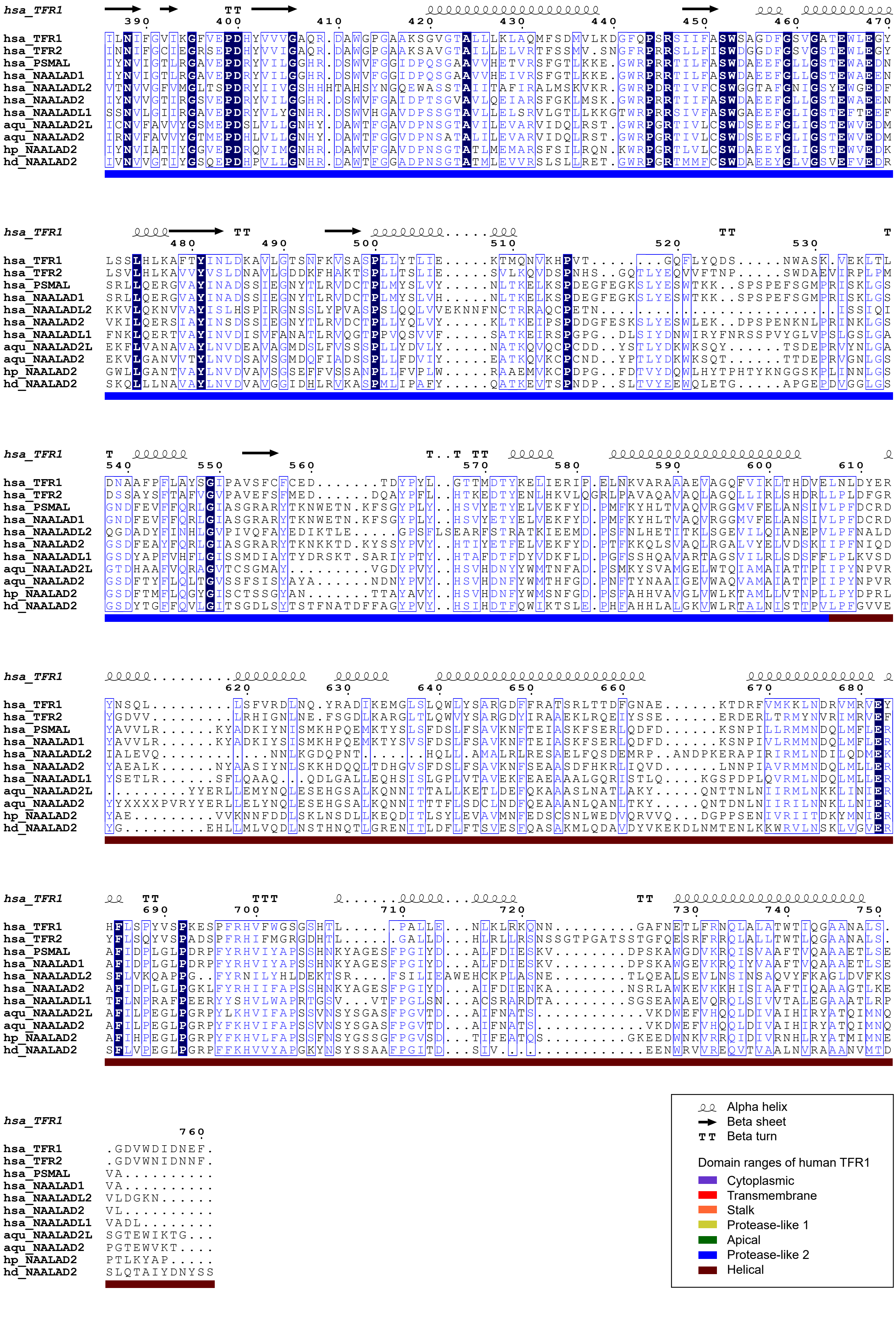
hsa_TFR1
hsa_TFR2
hsa_PSMAL
hsa_NAALAD1
hsa_NAALADL2
hsa_NAALAD2
hsa_NAALADL1
aqu_NAALAD2L
aqu_NAALAD2
hp_NAALAD2
hd_NAALAD2
Alpha helix
Beta sheet
Beta turn
Domain ranges of human TFR1
Cytoplasmic
Transmembrane
Stalk
Protease-like 1
Apical
Protease-like 2
Helical
