# Supplementary material for: Iron metabolic pathways in the processes of sponge plasticity
Source: PLoS One. 2020 Feb 21;15(2):e0228722. doi: 10.1371/journal.pone.0228722 (PMC7034838; doi:10.1371/journal.pone.0228722)
Supplement: S1 Table — (PDF) [file pone.0228722.s008.pdf]

S1 Table. Transrate report for transcriptome assemblies.

	H. dujardini	H. panicea
Contig metrics		
# of contigs	583,538	798,940
# of contigs with length <200	204	161
# of contigs with length >1000	88,154	109,739
# of contigs with length >10000	532	541
smallest contig length	178	178
largest contig length	125,246	45,348
mean contig length	691	643.54
N90	289	281
N70	513	466
N50	999	875
N30	2,145	1,839
N10	4,796	4,310
# of contigs with ORF	78,936	168,512
% of contigs with ORF	13.5%	21.1%
mean % of the contig length covered by the ORF	50.9%	63.1%
gc content	46.0%	43.0%
Read mapping (general)		
# of read pairs provided	291,295,519	146,912,616
# of read pairs mapped	271,666,726	129,064,589
# of read pairs mapping in a way indicative of good assembly	252,763,186	114,994,645

# of reads pairs mapping in a way indicative of bad assembly	18,903,540	14,069,944
# of potential links btwn contigs that are supported by the reads	371,972	298,252
Read mapping (absolute values)		
# bases in contigs	402,980,722	514,179,169
# of bases that are not covered by any reads	43,015,603	55,685,856
# of contigs that contain at least one base with no read coverage	361,090	401,368
# of contigs that have a mean per-base read coverage of < 1	27,267	33,998
# of contigs that have a mean per-base read coverage of < 10	28,706	408,726
# of contigs that have >=50% est. chance of being segmented	63,552	59,636
# of good contigs (optimizing assembly score)	291,557	420,248
Read mapping (%)		
% of the provided read pairs that mapped successfully	93.3%	87.9%
% of read pairs mapping in a way indicative of a good assembly	86.8%	78.3%
% of read pairs mapping in a way indicative of bad assembly	6.5%	9.6%
% of bases that are not covered by any reads	10.7%	10.8%
% of contigs that contain at least one base with no read coverage	61.9%	50.2%
% of contigs that have mean per-base read coverage of < 1	4.7%	4.3%
% of contigs that have mean per-base read coverage of < 10	4.9%	51.2%
% of contigs that have >=50% est. chance of being segmented	10.9%	7.5%
% of good contigs (optimizing assembly score)	50.0%	52.6%
TRANSRATE ASSEMBLY SCORE	0.17	0.19
