# Supplementary material for: Iron metabolic pathways in the processes of sponge plasticity
Source: PLoS One. 2020 Feb 21;15(2):e0228722. doi: 10.1371/journal.pone.0228722 (PMC7034838; doi:10.1371/journal.pone.0228722)
Supplement: S2 Table — (PDF) [file pone.0228722.s009.pdf]

## S2 Table. BUSCO report for transcriptome assemblies.

| Assembly | BUSCOs found | Database (total number of records) | | | | | | | |
|---|---|---|---|---|---|---|---|---|---|
| | | Eukaryotes (303) | | Metazoa (978) | | Bacteria (148) | | Fungi (290) | |
| *H. dujardini* | Complete | 295 | 97.36% | 899 | 91.92% | 135 | 91.22% | 266 | 91.72% |
| | Single-copy | 267 | 88.12% | 815 | 83.33% | 45 | 30.41% | 222 | 76.55% |
| | Duplicated | 28 | 9.24% | 84 | 8.59% | 90 | 60.81% | 44 | 15.17% |
| | Fragmented | 2 | 0.66% | 27 | 2.76% | 6 | 4.05% | 18 | 6.21% |
| | Missing | 6 | 1.98% | 52 | 5.32% | 7 | 4.73% | 6 | 2.07% |
| *H. panicea* | Complete | 300 | 99.01% | 954 | 97.55% | 146 | 98.65% | 280 | 96.55% |
| | Single-copy | 118 | 38.94% | 529 | 54.09% | 15 | 10.14% | 94 | 32.41% |
| | Duplicated | 182 | 60.07% | 425 | 43.46% | 131 | 88.51% | 186 | 64.14% |
| | Fragmented | 2 | 0.66% | 15 | 1.53% | 1 | 0.68% | 10 | 3.45% |
| | Missing | 1 | 0.33% | 9 | 0.92% | 1 | 0.68% | 0 | 0.00% |
