# Supplementary material for: Iron metabolic pathways in the processes of sponge plasticity
Source: PLoS One. 2020 Feb 21;15(2):e0228722. doi: 10.1371/journal.pone.0228722 (PMC7034838; doi:10.1371/journal.pone.0228722)
Supplement: S3 Table — (PDF) [file pone.0228722.s010.pdf]

S3 Table. Total number of single-end reads per sample for *H. dujardini* and their alignment rate reported by RSEM w.r.t. transcriptome assembly made of a separate set of paired-end reads (without decontamination).

**Sample type**	**Tissue**						**Cells**						**Aggregates**			
**Replicate**	**tiss1**		**tiss2**		**tiss3**		**cell1**		**cell2**		**cell3**		**aggr1**		**aggr2**	
**Total # of reads**	27,447,255	100.0%	30,413,614	100.0%	29,128,625	100.0%	30,841,015	100.0%	28,989,165	100.0%	31,557,272	100.0%	34,035,073	100.0%	28,905,672	100.0%
**Aligned 0 times**	1,411,557	5.1%	1,690,290	5.6%	609,521	2.1%	1,374,101	4.5%	1,297,912	4.5%	706,074	2.2%	797,282	2.3%	633,529	2.2%
**Aligned 1 time**	3,494,839	12.7%	3,903,454	12.8%	3,553,570	12.2%	3,701,160	12.0%	3,501,701	12.1%	3,269,077	10.4%	3,726,933	11.0%	3,192,484	11.0%
**Aligned >1 times**	22,540,859	82.1%	24,819,870	81.6%	24,965,534	85.7%	25,765,754	83.5%	24,189,552	83.4%	27,582,121	87.4%	29,510,858	86.7%	25,079,659	86.8%
