# Supplementary material for: Iron metabolic pathways in the processes of sponge plasticity
Source: PLoS One. 2020 Feb 21;15(2):e0228722. doi: 10.1371/journal.pone.0228722 (PMC7034838; doi:10.1371/journal.pone.0228722)
Supplement: S4 Table — (PDF) [file pone.0228722.s011.pdf]

S4 Table. Accession numbers for protein and mRNA (if it contains iron-responsive element) sequences of genes involved in iron metabolism and the response to hypoxia in *H. dujardini* and *H. panicea*, and accession numbers to their homologs / best blastp hits in *H. sapiens* and *A. queenslandica.*

Homologs / best blastp hits		Protein name	*H. dujardini*		*H. panicea*	
H. sapiens	*A. queenslandica*		Protein CDS	Full mRNA with IRE	Protein CDS	Full mRNA with IRE
NP_005680.1	XP_011404769.1	**ABCB6**	MK520974	—	BankIt2237851 Seq19 MN103208	BankIt2254648 Seq20 MN339471
NP_004290.2	XP_019851441.1	**ABCB7**	BankIt2296189 Seq2 MN867944	—	BankIt2296189 Seq4 MN867946	—
XP_005263412.1	XP_019854880.1	**ABCG2**	MK520972	—	BankIt2237851 Seq20 MN103209	—
NP_001089.1	XP_019850428.1	**ACO2**	BankIt2243561 Seq2 MN178306	BankIt2254648 Seq6 MN339457	BankIt2243561 Seq4 MN178308	—
NP_078970.3	XP_019848643	**ADGB**	BankIt2234598 Seq26 MN075826	BankIt2254648 Seq15 MN339466	BankIt2237851 Seq35 MN103224	BankIt2254648 Seq18 MN339469
NP_001357003.1	XP_011403685.2	**AKT**	BankIt2234598 Seq10 MN075812	—	BankIt2237851 Seq18 MN103207	—
XP_011516666.1	XP_019852250.1	**ALAD/hemB**	MK520963	BankIt2254648 Seq10 MN339461	BankIt2237851 Seq10 MN103199	—
NP_000679.1	XP_003383027.1	**ALAS**	MK520962	—	BankIt2237851 Seq4 MN103193	BankIt2254648 Seq21 MN339472

XP_016875158.1	XP_019857934.1	**ARNT**	BankIt2234598 Seq15 MN075817	—	BankIt2237851 Seq23 MN103212	—
XP_011524437.1	XP_003386092.1	**BCL2-like**	BankIt2234598 Seq7 MN075809	BankIt2254648 Seq4 MN339455	BankIt2237851 Seq15 MN103204	—
NP_001159.2	XP_003382947.1	**BIRC5/survivin**	BankIt2234598 Seq14 MN075816	BankIt2254648 Seq11 MN339462	BankIt2237851 Seq22 MN103211	—
XP_016883980.1	XP_003389391.1	**CBS**	BankIt2234598 Seq5 MN075807	—	BankIt2237851 Seq9 MN103198	—
NP_001008389.1	XP_003389059.1	**CDGSH/mitoNEET**	BankIt2234598 Seq24 MN075824	—	BankIt2237851 Seq33 MN103222	—
NP_000088.3	XP_003389974.2	**CPOX/hemF**	MK520967	—	BankIt2237851 Seq1 MN103190	—
NP_001893.2	XP_003387879.1	**CTH**	BankIt2234598 Seq6 MN075808	BankIt2254648 Seq16 MN339467	BankIt2237851 Seq13 MN103202	—
XP_005268968.1	XP_019849505.1	**DMT1**	MK520987	—	BankIt2237851 Seq30 MN103219	—
NP_001012533.1	XP_003385484.1	**FECH/hemH**	MK520970	—	BankIt2237851 Seq14 MN103203	—
NP_055400.1	XP_003386113.1	**FPN1/SLC40A1**	MK520986	—	BankIt2237851 Seq28 MN103217	—
NP_002023.2	XP_019854974.1	**FTH1**	MK520983	BankIt2254648 Seq1 MN339452	BankIt2237851 Seq3 MN103192	BankIt2254648 Seq17 MN339468
NP_002037.2	XP_003387153.2	**GAPDH**	BankIt2234598 Seq23 MN075823	—	BankIt2237851 Seq32 MN103221	—

XP_016856576.1	XP_003386865.1	**GST mu-like**	BankIt2234598 Seq1 MN075803	—	—	—
NP_000845.2	XP_003387275.2	**GST theta-like**	BankIt2234598 Seq2 MN075804	BankIt2254648 Seq9 MN339460	BankIt2237851 Seq5 MN103194	—
NP_542400.2	XP_011410503.1	**HCP1/SLC46A1**	BankIt2296189 Seq1 MN867943	—	BankIt2296189 Seq3 MN867945	—
NP_001521.1	XP_011403284.1	**HIFa/SIM-like 1**	BankIt2234598 Seq16 MN075818	—	BankIt2237851 Seq24 MN103213	—
XP_016883931.1	XP_011403286.2	**HIFa/SIM-like 2**	BankIt2234598 Seq17 MN075819	—	BankIt2237851 Seq25 MN103214	—
NP_851397.1	XP_019850799.1	**HIFa/SIM-like 3**	BankIt2234598 Seq18 MN075820	BankIt2254648 Seq12 MN339463	BankIt2237851 Seq26 MN103215	—
NP_060312.2	XP_011408917.1	**HRG1-like**	MK520971	BankIt2254648 Seq13 MN339464	—	—
NP_002188.1	XP_011404037.2	**IRP1/ACO1**	MK520985	—	BankIt2243561 Seq3 MN178307	—
NP_057696.2	XP_003389716.2	**MFRN/mitoferrin**	MK520973	—	BankIt2237851 Seq29 MN103218	—
XP_005263495.1	XP_019856828.1	**MTOR**	BankIt2234598 Seq10 MN075812	—	BankIt2237851 Seq18MN103207	—
NP_004467.1	XP_019850918.1	**NAALAD2**	BankIt2234598 Seq4 MN075806	BankIt2254648 Seq2 MN339453	BankIt2237851 Seq7 MN103196)	—
NP_005737.1	XP_003385030.1	**NAMPT**	BankIt2234598 Seq9 MN075811	—	BankIt2237851 Seq17 MN103206	—

NP_002493.3	NP_001266238.1	**NFKB1**	BankIt2234598 Seq8 MN075810	—	BankIt2237851 Seq16 MN103205	—
NP_067080.1	XP_003387899.1	**NGB**	MK520984	—	BankIt2237851 Seq31 MN103220	—
NP_001191142.1	XP_011410183.2	**NOS1**	BankIt2234598 Seq19 MN075821	BankIt2254648 Seq8 MN339459	BankIt2237851 Seq27 MN103216	—
XP_005271588.1	XP_003383634.1	**PBGD/hemC**	MK520964	BankIt2254648 Seq7 MN339458	BankIt2237851 Seq12 MN103201	—
NP_000300.1	XP_011405872.1	**PPOX/hemY**	MK520968	—	BankIt2237851 Seq2 MN103191	BankIt2254648 Seq19 MN339470
NP_060816.1	XP_019854307.1	**RSAD1/hemW**	BankIt2234598 Seq27 MN075827	—	BankIt2237851 Seq36 MN103225	—
NP_001013682.2	XP_011403770.2	**SDR2/FRRS1**	BankIt2234598 Seq25 MN075825	—	BankIt2237851 Seq34 MN103223	—
NP_001013454.1	XP_003389641.1	**TST/MPST**	BankIt2234598 Seq3 MN075805	—	BankIt2237851 Seq6 MN103195	—
NP_000365.3	XP_019855693.1	**UROD/hemE**	MK520966	BankIt2254648 Seq3 MN339454	BankIt2237851 Seq8 MN103197	—
XP_024303922.1	XP_019852312.1	**UROS/hemD**	MK520965	BankIt2254648 Seq14 MN339465	BankIt2237851 Seq11 MN103200	—
