# Supplementary material for: Iron metabolic pathways in the processes of sponge plasticity
Source: PLoS One. 2020 Feb 21;15(2):e0228722. doi: 10.1371/journal.pone.0228722 (PMC7034838; doi:10.1371/journal.pone.0228722)
Supplement: S5 Table — (PDF) [file pone.0228722.s012.pdf]

S5 Table. Features of iron-responsive elements (IREs) in *H. dujardini* and *H. panicea* mRNAs of genes involved in iron metabolism and hypoxic response, predicted by SIREs web-server [60].

Organism	SIREs Quality	Gene name	Motif location		CDS region		Motif region		Loop class	Upper stem features				RNAFold matches			Free energy, kcal/mol	Motif sequence
			Expected	Observed	Start	End	Start	End		Mismatches	3' bulge	N25	GU pairs	Loop	Pairs	C8 bulge		
H. dujardini	High	FTH1	5'	5'	89	598	4	35	1	-	-	C	1	☑	☑	☑	-7.4	AGTCTTG**C**TGTGC**CAGTGA**GTACACGGACCG
	High	NAALAD2 (TFR1 hom.)	3'	3'	53	2 428	2 964	2 995	18	7-25:A_A	-	A	1	☑	☑	☑	-4.9	CAGGGAA**G**CTTGA**CAGTGA**TCAGGAAGCATG
	Med	UROD/hemE		3'	66	1 145	1 298	1 329	2	10-23:T_T	-	T	0	☒	☑	☑	0	CAATACA**C**ATGTA**CAGAGA**TACTTTGAATAC
	Med	BCL2		3'	119	790	2 498	2 530	18	-	N22b:G	C	0	☑	☑	☒	-4.8	CGAGCCG**G**TGATC**CAGTGA**GATGCACAGCTGG
	Med	ACO2	5'	3'	80	2 431	3 532	3 563	8	10-23:A_C	-	C	0	☒	☑	☑	-2.1	TTGCCAG**C**CACTG**CAGGGA**CAGCGCTCCCGT
	Med	ACO2	5'	3'	80	2 431	4 530	4 561	2	9-24:C_T	-	T	2	☒	☑	☑	-2.7	GGCAGCA**C**CTCGT**CAGAGA**GCGGTTCTTCCT
	Med	HMBS/hemC		3'	5 115	6 143	6 451	6 483	15	-	N20b:A	A	1	☑	☑	☑	-4.4	CACGTCT**C**GCTGT**GAGAGG**GACAGCACTATGT
	Med	NOS1		CDS	144	4 574	3 616	3 647	16	11-22:A_C	-	C	1	☒	☑	☑	-5.4	CCAGCGG**C**GGAGG**GGGAGG**CCCCTCCAAGCT
	Low	GST (iso2)		3'	253	942	1 767	1 798	15	10-23:A_A	-	G	2	☒	☑	☒	0	GGAGTGT**C**TAGGA**GAGAGT**TTCAAGAAAGTG
	Low	ALAD/hemB		5'	1 277	2 254	992	1 023	6	10-23:A_A	-	G	2	☒	☑	☒	-1.7	ATTCGCT**C**TAGCT**CTTAGC**AGCAGGGAATTC
	Low	BIRC5/survivin		5'	1 796	2 269	1 411	1 442	8	-	-	T	1	☒	☑	☒	-3.5	GCACTGA**C**CTGCA**CAGGGC**TGTAGTGGCTGG
	Low	HIFa/SIM-like 3	5'	5'	3 184	5 052	1 544	1 575	15	11-22:A_G	-	A	2	☑	☑	☒	-2.2	GGGTCTT**C**TCAGG**GAGAGG**CTGGGAGACAGA
	Low	HRG1 (pred. hom.)		3'	197	772	4 866	4 897	19	7-25:C_T	-	T	2	☒	☑	☒	-3.8	TGCCTAC**C**TTGCC**AAGTTT**GGTAGTCCAAGT
	Low	HRG1 (pred.hom.)		3'	197	772	5 274	5 306	8	-	N21b:C	T	2	☒	☑	☑	0	TCTTGAG**C**TTTTT**CAGGGA**AACGAATAGACAA
	Low	HMBS/hemC		5'	5 115	6 143	3 472	3 503	18	12-21:T_C	-	T	1	☒	☑	☒	0	TAGCCTC**C**ACTGG**CAGTGG**CCAGAGCCTGGG
	Low	UROS/hemD		CDS	287	1 084	545	576	4	13-20:C_A	-	G	2	☑	☑	☒	-5.5	TGGTCGT**C**TGTGC**CCGTGT**ACGCAGTGGGCG
	Low	NOS1		CDS	144	4 574	4 003	4 034	2	11-22:C_A	-	G	1	☒	☑	☒	-8.4	ACCTGGC**C**CTCTC**CAGAGA**GGAAGGCCAGCC

	Low	ADGB		CDS	485	4 903	1 429	1 460	15	11-22:T_C	-	G	0	☒	☑	☒	-6.8	CCACGCC**C**CCTCC**GAGAGG**GGCGGGTCCGGG
	High	CTH		CDS	1 268	2 422	1 872	1 903	1	9-24:G_G	-	T	1	☑	☑	☑	-5.0	ACCTCAA**C**GGACA**CAGTGA**TGTTGTGATGGG
H. panicea	High	FTH1	5'	5'	107	625	6	37	1	-	-	C	0	☑	☑	☑	-6.4	TGCTTTG**C**TACGT**CAGTGA**ACGTACGGGCTG
	Med	ADGB		5'	1 102	5 067	1 025	1 056	16	12-21:G_G	-	T	0	☒	☑	☑	-1.2	ATGACAA**C**TAGGT**GGGAGG**AGCTATACAGGA
	Med	ADGB		CDS	1 102	5 067	2 830	2 861	14	9-24:A_C	-	T	0	☒	☑	☑	-1.3	ACTGGCA**C**AAAAG**GAGCCA**CTTTCTCACTAC
	Med	PPOX/hemY		CDS	220	1 650	1 595	1 626	3	13-20:A_G	-	A	0	☒	☑	☑	-5.9	TCAGTGT**C**AATGA**CTGTGT**GCATTATGCTGT
	Low	ABCB6		CDS	320	2 983	1 413	1 444	19	7-25:A_G	-	G	2	☑	☑	☒	-0.5	TGAACAA**C**ATGAG**AAGTTA**CTTGTGGATCAC
	Low	ALAS		5'	308	2 146	44	75	19	9-24:A_G	-	T	1	☑	☑	☒	0	CACGAGA**C**ATTTT**AAGTTC**AAAGGTCACTTT
